# Experimental verification of the six sectors neural DTC approach of squirrel cage induction motors

**DOI:** 10.1038/s41598-025-95333-y

**Published:** 2025-03-28

**Authors:** Abdessmad Milles, Habib Benbouhenni, Noureddine Bensedira, Nicu Bizon, Naamane Debdouche, Ilhami Colak, Ghoulemallah Boukhalfa, Z. M. S. Elbarbary, Mohammed M. Alammar

**Affiliations:** 1 Department of Electromechanics, Faculty of Sciences and Technology, Laboratory of Materials Physics, Radiation and Nanostructures (LPMRN), University of Bordj Bou Arréridj, 34265 Bordj Bou Arréridj, Algeria; 2 Department of Electrical Engineering, LAAS Laboratory, National Polytechnic School of Oran- Maurice Audin, BP 1523 Oran El M’naouer, Oran, Algeria; 3 University of Batna 2, Batna, Algeria; 4 Faculty of Electronics, Communication and Computers, National University of Science and Technology POLITEHNICA Bucharest, Pitești University Center, 110040 Pitesti, Romania; 5 Brothers Mentouri University, Constantine, Algeria; 6 Department of Electrical and Electronics Engineering, Istinye University, Istanbul, Turkey; 7 Department of Electrical Engineering, College of Engineering, King Khalid University, KSA, P.O. Box 394, 61421 Abha, Saudi Arabia; 8 Center for Engineering and Technology Innovations, King Khalid University, 61421 Abha, Saudi Arabia

**Keywords:** Squirrel cage induction machines, Direct torque control, Neural networks, Experimental work, Energy science and technology, Engineering

## Abstract

The direct torque control (DTC) approach is one of the suitable solutions for controlling squirrel cage induction machines (SCIMs) due to its distinctive performance compared to other strategies and its simplicity. However, using this approach has several drawbacks and problems. This paper presents an experimental work using real equipment of an innovative method that combines six sectors of DTC technique and neural networks (NNs). The use of an NN algorithm allows for overcoming problems of the DTC approach, such as reducing torque ripples. Using the NN technique, the operation of the SCIM inverter is controlled, as the NN technique provides the pulses necessary to run the inverter, which allows for improving the quality of the current. Therefore, the presented approach is based on the usual method, using the same estimation equations. First, the validity of the designed approach was tested using MATLAB, comparing the results with the DTC approach. The results obtained showed a high ability of the six sectors’ NN-DTC approach to significantly enhance the quality of torque and current, which confirms the competence of using NNs. Secondly, real equipment was used to verify the simulation results and the extent of the efficiency and competence of the six sectors NN-DTC approach compared to the DTC technique in terms of improving the quality of current and torque. These experimental results obtained are of great value in the field of control, as they give a clear picture of the advantage of the six sectors of the NN-DTC approach in improving the features of the control system, which makes it more suitable for different applications in the future.

## Introduction

Electrical machines are many and varied, and they can be divided according to the type of current into direct-current electric machines and alternating-current electric machines^1^. Nowadays, alternating current machines are the most widely used and widespread machines in many applications such as traction and the field of renewable energies. These alternating current machines can be classified into induction machines (IMs)^2^ and synchronous machines^3^, where the difference between them lies in operation, cost, ease of control, maintenance, and robustness^4^. As is known, IMs are characterized by greater robustness, lower cost, less maintenance, and ease of control compared to other machines, which makes them widely used and of great importance in industrial applications^5^. In^6^, IM was used in a water turbine power system as a load. The IM is connected using AC-DC-AC converters. The use of these converters allows for good IM feeding and operation.

The squirrel cage IM (SCIM) is one of the most popular and sought-after types of IMs around the world due to its many advantages such as durability and less maintenance. In addition to ease of control and low costs^7^.

In the industrial field, the availability of SCIM in production lines is mandatory to ensure continuity of service and good performance. To avoid malfunctions in parts of this machine, it is necessary to use a powerful control that is described by high competence and great robustness. As is known, any malfunction in the machine may lead to a complete cessation of the production process, leading to increased loads and huge financial losses, which are undesirable^8^.

In^9^, the approach field-oriented control (FOC) technique was used to control SCIM. This algorithm relies on the use of a proportional-integral (PI) regulator to control the feature quantities. This approach is described by a fast dynamic response (DR) and ease of realization, but it is affected by changes in the machine parameters, which causes an augment in torque and current ripples. Another approach that has been proposed to control the speed of SCIM in^10^ is the direct torque control (DTC) technique. This approach is different from the FOC technique in the principle and results provided. The DTC approach relies on the use of hysteresis comparators (HCs) to regulate the distinct amounts. Also, it relies on the use of a switching table (ST) to produce the pulses needed to operate the machine inverter. Therefore, this algorithm is described as inexpensive, quick DR, simple, easy to realize, and contains few gains^11^. The DTC technique relies on estimating the characteristic quantities, which makes it affected by changes in the machine’s parameters. This effect results in an increase in torque fluctuations and the total harmonic distortion (THD) of the current. In the work^12^, the author compared the performance of the DTC technique compared to the FOC strategy of the IM strategy. First, the mathematical model (MM) for each strategy was given, mentioning the advantages of each approach. Secondly, similarities and differences between the two approaches are given. These two strategies were implemented using MATLAB. The results obtained showed the superiority of the DTC approach over the FOC approach in terms of reducing torque and flux ripples. These results highlight the superiority of the DTC approach, making it of interest in the control field. To minimize torque fluctuations in SCIM, nonlinear approaches such as the backstepping control (BC) technique^13^ and sliding mode control (SMC)^14^ were used, as these approaches are characterized by high competence and great durability. The use of these approaches depends on the use of the pulse width modulation (PWM) technique to control the action of the inverter. This approach increases the complexity of the SCIM control system and the difficulty of implementing it. The use of these approaches depends on knowing the SCIM model accurately, which makes the torque quality affected in the event of a defect. Also, using these approaches increases the number of gains, which makes it difficult to adjust the DR, which is a negative.

One of the solutions that have been proposed for controlling SCIM is to combine several different approaches to minimize torque fluctuations and augment the robustness of the control system, such as combining the BC technique and synergetic-SMC controller^15^. This algorithm is described by high efficacy and great robustness. This is demonstrated by all the tests completed, as the torque ripples and THD values were significantly reduced. Also, the values ​​of overshoot and steady-state error (SSE) of torque, flux, and speed have been reduced compared to the classical approach. However, despite this high competence, the designed algorithm is described by several cons, including the presence of a significant number of gains, which makes it difficult to adjust the DR with ease. Also, the complexity and difficulty of realization are among the most prominent cons of this approach, which makes it expensive. Fuzzy logic (FL) and the SMC strategy were combined to obtain a robust and reliable strategy for controlling IM^16^. The FL approach was used to compensate for the Sign(U) function of the SMC technique. Therefore, the FL-SMC approach is characterized by high performance, great efficiency, and high robustness compared to the traditional approach. The use of the FL-SMC approach allows for reducing torque ripples and reducing the severity of chattering. However, using the FL-SMC approach has its drawbacks, which lie in the lack of a rule that facilitates the use of the FL strategy, as reliance is placed on experimentation and simulation to determine the best number of FL rules.

In the work^17^, both the FL technique and neural networks (NNs) were used to get better the competence and effectiveness of the DTC approach of SCIM. In this work, NN was used to compensate for ST and thus create the pulses needed to control the SCIM inverter. Also, the FL was used to compensate for the PI of the speed regulator and thus generate the torque reference value. Therefore, the approach is described by great efficiency, high ability to reduce ripples, and undershoot of torque and speed. The negative of this approach lies in its reliance on estimating flux and torque, which makes it affected by changes in SCIM parameters and this appears in the rise in torque fluctuations and the THD value of current in the robustness test. Moreover, no rule facilitates the use of both NN and FL, which makes it more difficult to obtain good results. In^18^, a new DTC approach for SCIM was proposed, based on the use of the model predictive control (MPC) technique to get better quality of current and torque. This approach is different from the DTC approach, as experimental work was used to implement it and verify its performance and robustness. The results of the MPC-DTC approach were compared with the DTC approach, where the experimental results showed that the MPC-DTC approach can significantly enhance the torque fineness while increasing the robustness of the control system. However, despite this competence, the MPC-DTC technique is described by complexity and difficulty of implementation. A new strategy for DTC was proposed in the work^19^ based on the use of the genetic algorithm. In this proposed strategy, a genetic algorithm was used to calculate the gain values ​​of the PI controller used to determine the reference value of the voltage. The proposed strategy relies on the use of both ST and HC. The proposed strategy is characterized by simplicity, ease of implementation, and quick DR. This proposed strategy was applied to a double-fed IM, where MATLAB was used to implement it and verify its effectiveness compared to the strategy based on the PI controller. Simulation results showed the effectiveness and efficiency of the proposed approach based on the genetic algorithm in improving torque ripples and the DR to both speed and torque. The strategy based on the genetic algorithm reduces response time and overshoot speed by rates estimated at 57.14% and 90%, respectively, compared to the traditional DTC approach. Also, the strategy based on the genetic algorithm reduces the values ​​of response time, ripples, and overshoot of torque by percentages estimated at 63.49%, 64.44%, and 86.677%, respectively, compared to the traditional DTC approach. Despite these large percentages presented by the proposed approach, the problem of ripples remains an issue due to the proposed approach’s reliance on HC-type controllers in order to control torque and flux. Grey wolf optimization (GWO) is the solution proposed in^20^ to overcome the problems and drawbacks of the DTC strategy of dual star IM. In this work, the GWO strategy was used to calculate the gain values ​​of the PI of the speed controller. The DTC-GWO strategy is a development and modification of the DTC strategy. The DTC-GWO strategy uses ST to control the operation of the machine inverter, and HC-type controllers are used to control torque and flux. Using both ST and HC makes the DTC-GWO approach less efficient if the system parameters change. The DTC-GWO strategy was compared to the DTC approach using MATLAB. The simulation results showed that the use of the DTC-GWO strategy allows significant improvement the value of torque and flux ripples compared to the DTC approach. Also, using the DTC-GWO approach significantly improves the DR to torque and speed compared to the traditional DTC technique. However, the DTC-GWO approach has disadvantages that lie in the use of torque and flux estimation, which makes it affected by changing machine parameters. Also, it is observed that there are ripples at the torque and current levels in the case of using the DTC-GWO approach, which is undesirable, and requires searching for a control strategy with a great ability to reduce the torque and current ripples. In^21^, a new DTC strategy based on the use of the FL technique is proposed. In this proposed strategy, the FL technique was used to compensate for the use of the PI of the speed controller. This proposed strategy was used to control the operation of double-fed IM. This proposed strategy is characterized by high durability, distinctive performance, quick DR, and great effectiveness in reducing torque fluctuations. In order to embody the mysterious Controller, 49 rules were used. The proposed strategy was implemented in a MATLAB environment with results compared to the DTC technique. Simulation results showed that the proposed approach significantly reduces torque ripples compared to the DTC approach. Also, the proposed approach improves the dynamic torque response significantly compared to the DTC approach. However, this proposed approach has drawbacks, namely that it is affected by the change in machine parameters as a result of its use of the DTC technique to control torque and flux.

Another experimental work on the DTC approach was carried out in^22^, where a new hybrid FL regulator was used to defeat the drawbacks of the DTC approach of SCIM. In this work, several different controls were proposed, namely PI-type FL control (FLC), a combination of PD-type FLC and I control, and a proportional-derivative (PD) type FLC technique. These proposed controllers were compared with the PI control. The experimental results show the advantages and disadvantages of these controllers and their ability to get better the fineness of torque and current compared to using a traditional regulator. The negative of these controls lies in the use of the FL approach which relies heavily on experience, and there is no rule to help apply it. Also, the use of these controls depends on the use of estimation of both torque and flux, which makes the proposed approach affected by changing machine parameters. The MPC approach based on the flux weakening strategy is the solution proposed in^23^ to get a better the characteristics of the DTC approach of SCIM. Using this algorithm significantly improved the characteristics of the control system. This algorithm is characterized by high competence, great robustness, and effectiveness in minimizing torque fluctuations, undershooting, and the THD value of the current. However, this approach is characterized by complexity, which is a negative thing that increases the difficulty of implementation and raises costs. In^24^, interval type-2 FL was used as an effective solution to overcome the low robustness of the DTC approach of SCIM, where the traditional controllers and ST were dispensed with and replaced with the proposed controller and the PWM approach. In this work, a five-level diode-clamped inverter was used to feed the SCIM, as using this inverter allows to increase the quality of the current and thus improve the quality of torque and flux. This approach is characterized by complexity, difficulty to realize, and expensive compared to the usual approach. However, experimental results highlight the advantage of this approach in terms of enhancing torque fineness and minimizing the THD of the current. Another solution proposed in^25^ is based on using a 12-sided polygonal voltage space vector to enhance the features of the DTC approach of SCIM. This solution differs from the conventional approach, as it is a development of the DTC. The DTC approach uses a flux vector for the sector identification and then the switching vector (SV) to control torque and flux. However, the designed DTC approach selects SVs based on the sector information of the estimated fundamental stator voltage vector (SVV) and its relative position concerning the stator flux vector (SFV). The designed DTC algorithm uses the exact positions of the primary SVV and the SFV to determine the optimal SV for fast torque control with small stator flux variation within the hysteresis range. The current DTC system allows full torque control with fast transient response at very low operating speeds while minimizing switching frequency variation. Empirical results showed high competence of the designed approach compared to the DTC approach in terms of reducing torque fluctuations and improving speed characteristics. Compared to the DTC technique, this approach is complex and difficult to implement, which negatively increases costs. Also, this proposed algorithm depends on estimating both flux and torque, which makes it affected by changing SCIM parameters, which causes a decrease in the fineness of the current and thus an augment in torque fluctuations. In^26^, the IM was used in photovoltaic water pumping systems with a robust control proposal. Water pumping systems using renewable energies are increasingly important as a renewable energy solution in rural areas, providing energy independence, cost savings, and environmental friendliness. This proposed energy system contains two main control units, where the maximum power point tracking based on NN controller is used to maximize energy extraction from the photovoltaic array by controlling the operating ratio of the DC-DC boost converter. In the second controller, the DTC-ANN strategy is used to regulate the operation of the IM through the switching pulses of the voltage source inverter. These two controllers play an essential role in the system, which increases the efficiency and performance of the considered control system. A neural approach was used to improve the power generation system based on photovoltaic cells and improve the effectiveness and efficiency of the pump. The strategy used to control the pump depends on the use of NNs in the speed controller, HC of torque and flux, and ST. Accordingly, three neural controllers were used to improve the characteristics of the DTC strategy used to control the operation of the pump. The proposed control system was implemented in the MATLAB environment using experimental work. Experimentally, the dSPACE DS1104 Board was used to verify the effectiveness and robustness of the proposed approach compared to the traditional approach. The results showed that using the neural approach allows improving flux ripples by an estimated 75.51% compared to the traditional DTC approach. Also, torque ripples were improved by an estimated 77.5% compared to the traditional DTC approach. Also, the results showed that using the neural approach allows improving the time response by an estimated 44.79%. These percentages highlight the effectiveness of using the neural approach in both the DTC strategy and the maximum power point tracking technique, making it a reliable solution in the field of control. In^27^, the author experimentally compares the DTC approach with the finite set model predictive control (FS-MPC) strategy for an IM. First, an MM was given for each control strategy, mentioning the advantages of each approach. Second, the two strategies were implemented using MATLAB under different operating conditions. Simulation results showed the superiority of the FS-MPC approach over the DTC approach in terms of improving flux and torque ripples. Real tools were used to verify the behavior of each approach. Experimental results showed the superiority of the FS-MPC approach over the DTC approach in terms of improving machine characteristics, and this is demonstrated by torque and flux ripples. However, although the FS-MPC approach is superior in terms of performance, the DTC approach excels in terms of simplicity, ease of implementation, and dynamic torque response. A robust predictive direct torque control approach was proposed in the work^28^ to compensate for the use of the DTC strategy. This proposed strategy is a development and modification of the DTC approach, where both ST and HC are compensated by a predictive block based on an optimization algorithm. Also, a robust predictive speed loop regulator was used instead of using a PI controller. Therefore, the proposed approach is characterized by high performance and great robustness. However, compared to the DTC approach, the proposed approach is complex, expensive, and difficult to implement. The proposed approach was implemented using the OPAL-RT platform under different working conditions, with the results compared to the traditional DTC approach. The results indicate that the proposed approach outperforms the conventional approach in terms of rejecting disturbances, showing robustness to parameter changes, and reducing torque ripple. In^29^, it is proposed to use an adaptive nonlinear DTC approach to control SCIM. This proposed technique is a modification of the DTC technique, as it relies on the use of a sliding-mode rotor-flux observer. Therefore, this designed approach is described by its high robustness and its great ability to enhance torque quality, as demonstrated by the results of empirical and simulation work. The disadvantages of this proposed approach are that it is difficult to realize, expensive, and has a greater degree of complexity than the DTC approach. In the work^30^, NNs were used to defeat the cons of the DTC technique of dual SCIM. In this work, a single inverter controlled by DTC technique based on NNs was used to power two motors with the same characteristics. The work done is of great importance as it provides an effective solution to reduce costs in the industrial field. The designed approach is described by high robustness and distinctive efficacy, which is demonstrated by reducing torque fluctuations and the THD value of the current, which is demonstrated by simulation results in all tests. The reliance of this approach on estimating torque and flux is affected by changes in machine parameters, which is negative. Also, no rule facilitates the use of NN techniques and obtaining good results.

The space vector modulation (SVM) technique is one of the approaches used to control the operation of the inverter, as it was relied upon in^31^ to defeat the disadvantages of the DTC technique of SCIM. The SVM approach was used to replace ST, and the traditional regulators were dispensed with and replaced with a PI regulator, where two regulators were used to control torque and flux. Also, to estimate the speed, a stator current error-based model reference adaptive system (MRAS) was used, which makes the system more performant and efficient. This proposed approach is characterized by high robustness and its ability to enhance torque quality, as the experimental results showed the extent of its ability to minimize torque fluctuations and the THD value of the current. Complexity, high cost, DR, and difficulty of realization are all drawbacks of this proposed approach compared to the DTC technique. The DTC approach gave a much better time for both flux and speed than the DTC technique. Another experimental work deals with the use of the sensorless DTC-SVM strategy in^32^, where this approach was applied to the matrix converter used to feed SCIM. In this work, the flux error and torque are geometrically combined into a new flux leakage vector to generate a deadwise constant control voltage vector. A new sensorless method was derived to estimate flux, stator resistance, rotor speed, and rotor resistance, and this proposed approach was verified experimentally using real instruments. In this work, the error model is used to transform the condition estimation problem into a parameter estimation problem assuming that the rotor speed is constant. Lyapunov’s theory was used to verify the stability of the algorithm, as the empirical results demonstrated the efficacy, performance, and robustness of the designed method in enhancing system features. The negative of this algorithm lies in the complexity and difficulty of realization, which searches for the best approach to obtain good results in terms of the quality of torque and current for SCIM. According to the work done in^33^, the DTC technique that uses 60° sectors of both flux and voltage vectors limits the quality of the current and torque of the SCIM. Therefore, it is necessary to use another approach that can enhance the fineness of torque and current, as it has been proposed to use a 12-sided polygonal space vector to overcome the disadvantages of the DTC approach. He designed an algorithm that utilizes twelve 30° sectors of both voltage vectors and flux to augment the degrees of freedom for the selection of proper vectors and minimize the torque fluctuation. This algorithm was implemented experimentally using real equipment, comparing the competence to the DTC technique. The empirical results showed that using the approach based on the 12-side polygonal space vector has a great ability to minimize torque fluctuations, undershoot, and SSE of flux and torque compared to the DTC approach. The negative of this approach lies in its effect on changing the machine parameters, which allows the quality of current and torque to decrease, which is undesirable. This effect can be attributed to the use of estimation of discriminant quantities. Another approach for the DTC technique of SCIM was proposed in^34^ based on the use of model predictive torque control to augment competence and effectively minimize torque and current fluctuations. This algorithm relies on the use of an integral super-twisting SMC (ISTSMC) technique to compensate for the use of a PI controller of speed. Also, a sliding mode speed observer was used to defeat problems associated with mechanical sensors. This approach was realized using MATLAB and was compared with both benchmark PI regulator and SMC technique using several different tests. The results showed that the proposed approach can significantly enhance the features of the control system compared to the other two approaches. However, the designed algorithm has disadvantages that lie in complexity, a significant number of gains, expensive, and difficult to achieve. In^35^, a new approach for the DTC technique of SCIM is proposed based on the use of both SVM and SMC techniques. In this experimental work, the SMC approach was used to command torque and flux, where the outputs of this regulator are the voltage reference values (VRVs). Also, the SVM technique was used to convert the VRVs ​​produced by the SMC technique into pulses to run the SCIM inverter. Therefore, the proposed approach is a development of the DTC technique, as simulation and experimental work were used to verify its validity while comparing the results with the DTC technique. The experimental results confirm the results and the ability of the designed approach to reduce torque fluctuations and augment robustness compared to the DTC approach. The negative of this approach lies in its use to estimate flux and torque, which makes it affected by changing machine parameters. In^36^, fractional-order proportional–integral–derivative (PID) controllers were used to improve the performance and effectiveness of the DTC approach of the IM. In this proposed approach, the particle swarm optimization (PSO) strategy was used to calculate the gain values ​​of the FO-PID controller. Using the PSO strategy allows for improving the characteristics of the proposed control and increasing its robustness. This proposed approach was implemented using MATLAB, and the results showed the superiority of the FOPID-PSO controller over the PI controller in terms of reducing torque and current ripples. The adaptive fractional-order sliding mode (FO-SM) control approach is the solution proposed in^37^ to overcome the problems of the DTC strategy of the IM. A FO-SM type control was used to control the flux and torque, and the outputs of these controls are voltage reference values. These VRVs ​​are converted into pulses to operate the machine inverter using the SVM strategy. Therefore, the DTC-SVM approach based on FO-SM controllers is characterized by high performance, great durability, and fast DR. But in terms of the degree of complexity, this approach is characterized by greater complexity and difficulty of completion compared to the traditional DTC technique. Also, the DTC-FO-SM approach has a significant number of gains which makes tuning the DR difficult compared to the conventional approach. Simulation results show the superiority of the DTC-FO-SM approach over the traditional approach in terms of reducing torque and current ripples. Also, in the case of changing machine parameters, the DTC-FO-SM approach gave good results compared to the traditional approach. In^38^, it was proposed to use the DTC strategy based on the three-level inverter with a six sector torque controller. This proposed strategy has been compared to the traditional approach using MATLAB. The simulation results highlighted the superiority of the proposed approach in terms of reducing torque ripples. But compared to the traditional approach, the proposed approach is complex and expensive, which is a negative.

This work deals with other experimental work on the 6-sector DTC technique of SCIM using NN approaches to defeat the problems of the 6-sector DTC technique. In this work, NNs were used to compensate for both ST and HC. Therefore, the first major contribution lies in the experimental verification of the 6 sectors NN-DTC technique of SCIM. The second major contribution of using NNs lies in alleviating the shortcomings of the 6 sectors DTC technique. First, the 6 sectors’ NN-DTC technique was verified using MATLAB, comparing the results to the 6 sectors’ DTC technique, where some tests were used to verify the ability, competence, performance, and robustness of the NN-DTC technique. The 6-sector NN-DTC approach has several advantages such as ease of realization, a small number of gains, simplicity, and fast DR. The proposed NN-DTC technique is a development of the 6 sectors DTC technique, as it differs from it in terms of performance and robustness. However, the designed approach uses the same estimation equations used in the 6-sector DTC technique. Second, the proposed approach was implemented experimentally using real equipment. This strategy was confirmed experimentally using several different tests and was experimentally compared to the 6 sectors DTC technique. The simulation results highlight the significant superiority of the 6 sectors NN-DTC technique and the importance of its use. Accordingly, the objectives achieved from this work can be extracted in the form of the following points:

- Experimentally verifying the proposed and 6 sectors DTC approach.
- Overcoming problems of 6 sectors DTC approach.
- Reducing torque and current fluctuations.
- Reduce the THD value of the current significantly compared to the traditional approach.
- Increasing the robustness and competence of the SCIM control system.

The sections of the article are as follows: The second section deals with the machine model. In the third section, the 6 sectors DTC approach are discussed in detail, mentioning the pros and cons. Six sectors neural DTC approach are listed in Section IV. In Section V, the simulation of the NN-DTC using MATLAB is discussed. In the sixth section, the designed approach was implemented using empirical work, where real tools were used to implement it. In Section VII, all conclusions of the work are listed.

## SCIM model

Traditionally, SCIM is one of the most popular and widespread machines in industrial applications due to its high durability and ease of control. Also, this machine has a significant yield that makes it an effective solution in industrial applications. Compared to the synchronous machine, SCIM does not require regular maintenance and is less expensive, which is a good thing. To use SCIM in this work, we first consider the MM using the Concordia transform. Using this conversion, equations are given for each part of the SCIM. Equation (1) represents the voltages of the SCIM, where these voltages are related to both flux and current. To control these voltages, it is enough to control the current or flux^39^.

1 $$\left\{\begin{array}{c}{V}_{\alpha s}={R}_{s}{I}_{\alpha s}+\frac{d}{dt}{\Phi }_{\alpha s}\\ {V}_{\beta s}={R}_{s}{I}_{\beta s}+\frac{d}{dt}{\Phi }_{\beta s}\\ {V}_{\beta r}={R}_{r}{I}_{\beta r}+\frac{d}{dt}{\Phi }_{\beta r}-w{\Phi }_{\alpha r}\\ {V}_{\alpha r}={R}_{r}{I}_{\alpha r}+\frac{d}{dt}{\Phi }_{\alpha r}+w{\Phi }_{\beta r}\end{array}\right.$$

To calculate the voltage on the axis (αβ), it is sufficient to use Eq. (2).

2 $$\left[\begin{array}{c}{V}_{\alpha s}\\ {V}_{\beta s}\end{array}\right]=\frac{2}{3}\left[\begin{array}{ccc}1& -1/2& -1/2\\ 0& \sqrt{3}/2& -\sqrt{3}/2\end{array}\right]\left[\begin{array}{c}{V}_{sa}\\ {V}_{sb}\\ {V}_{sc}\end{array}\right]$$

To express rotor currents in the axis (αβ), Eq. (3) can be used. This equation was obtained using Eq. (1), where the rotor voltage is zero for this machine^40^.

3 $$\left\{\begin{array}{c}{I}_{\alpha r}=\frac{{\Phi }_{\alpha s}-{L}_{s}{I}_{\alpha s}}{{M}_{sr}}\\ {I}_{\beta r}=\frac{{\Phi }_{\beta s}-{L}_{s}{I}_{\beta s}}{{M}_{sr}}\end{array}\right.$$

Equation (4) is considered for the flux of the rotor part in the αβ axis. This flux is related to the current.

4 $$\left\{\begin{array}{c}{\Phi }_{\alpha r}=\frac{{L}_{r}}{{M}_{sr}}({\Phi }_{\alpha s}-\sigma {L}_{s}{I}_{\alpha s})\\ {\Phi }_{\beta r}=\frac{{L}_{r}}{{M}_{sr}}({\Phi }_{\beta s}-\sigma {L}_{s}{I}_{\beta s})\end{array}\right.$$

Equation (4) can be written according to Eq. (5).

5 $$\left\{\begin{array}{c}\frac{d{\Phi }_{\alpha r}}{dt}=\frac{{L}_{r}}{{M}_{sr}}(\frac{d{\Phi }_{\alpha s}}{dt}-\sigma {L}_{s}\frac{d{I}_{\alpha s}}{dt})\\ \frac{d{\Phi }_{\beta r}}{dt}=\frac{{L}_{r}}{{M}_{sr}}(\frac{d{\Phi }_{\beta s}}{dt}-\sigma {L}_{s}\frac{d{I}_{\beta s}}{dt})\end{array}\right.$$

Equation (6) represents the torque of the SCIM used in this experimental work ^41^.

6 $${T}_{e}=\frac{p{M}_{sr}}{{L}_{r}}({\Phi }_{\alpha r}{I}_{\beta s}-{\Phi }_{\beta r}{I}_{\alpha s})$$

Equation (7) shows the relationship between speed and torque. Through this relationship, the operation of the SCIM and the rotational speed can be controlled.

7 $$J\frac{d{\Omega }_{r}}{dt}={T}_{e}-{T}_{r}-{f}_{r}{\Omega }_{r}$$

To control the operation of this machine, several different approaches have been used. The most prominent of these approaches used is the 6-sector DTC approach, which will be discussed in the next section.

## Six sector DTC approach

In this section, the 6-sector DTC approach is discussed in detail, mentioning the negatives and positives. This approach is different from the FOC approach, as it is considered a linear approach that has a quick DR^42^. It is also characterized by ease of implementation, inexpensive, and easy to apply to simple and complex systems. Using this approach does not require data on the MM of the system, as simple controls are used to control torque and flux^43^. These controllers do not require complex calculations, as a three-level HC is used to control torque and a two-level HC is used to control flux.

**Fig. 1:**
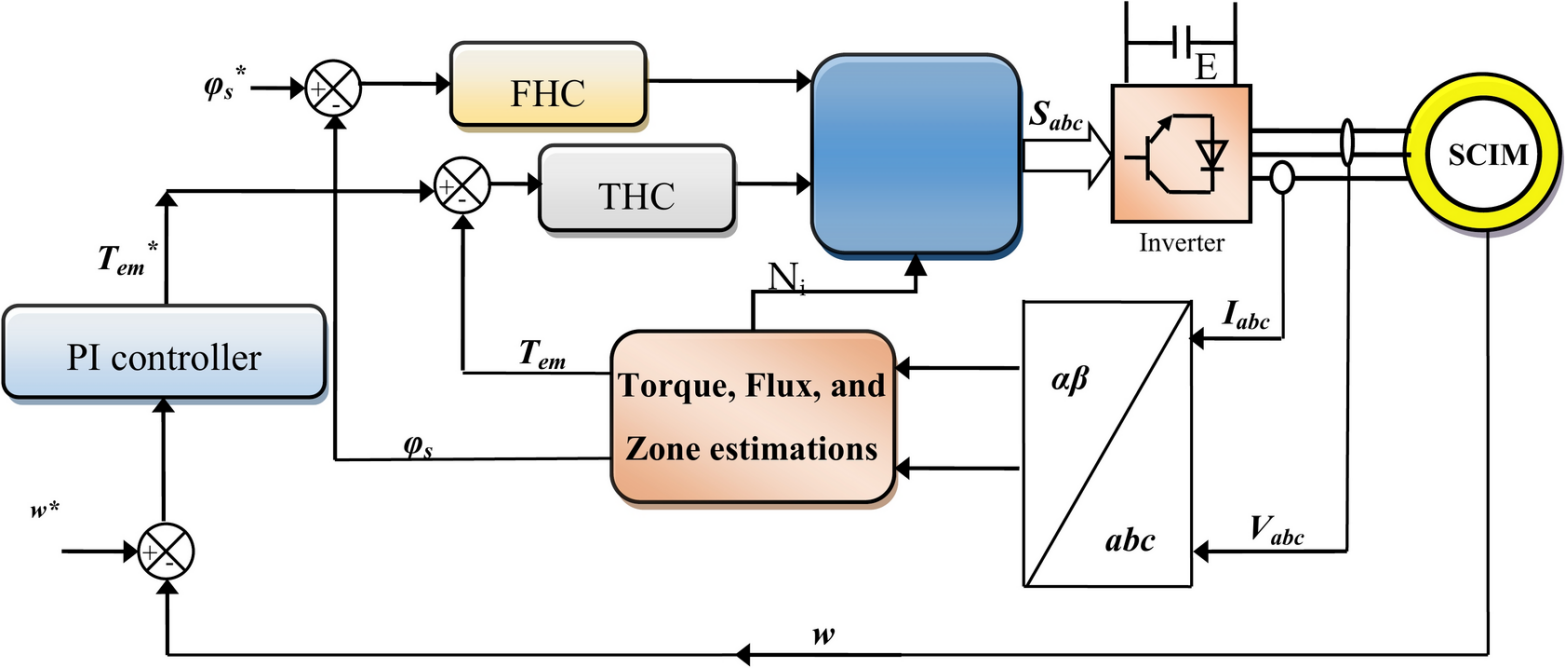
Six sectors DTC approach of SCIM.

Figure 1 represents the six sectors DTC approach of SCIM. In this approach, a PI-type regulator is used to determine the torque reference value based on the speed line. Also, this approach relies on estimating torque, sectors, and flux. To create the pulses needed to run the inverter, the ST is used, as it has three inputs and three outputs. These outputs are the operating pulses of the SCIM inverter.

To extract inverter operating pulses, Table 1 is used. This table gives the necessary voltage values ​​according to the condition of both the HC (torque (*T*_*e*_) and flux) and the sector. In this table, there are only 6 sectors^43^.

**Table 1:** The ST of 6 sectors DTC approach.

N_i_		1	2	3	4	5	6
HC_Te_	**HC**_**flux**_						
1	**1**	2	3	4	5	6	1
	**0**	1	2	3	4	5	6
	**–1**	6	1	2	3	4	5
0	**1**	3	4	5	6	1	2
	**0**	4	5	6	1	2	3
	**–1**	5	6	1	2	3	4

**Fig. 2:**
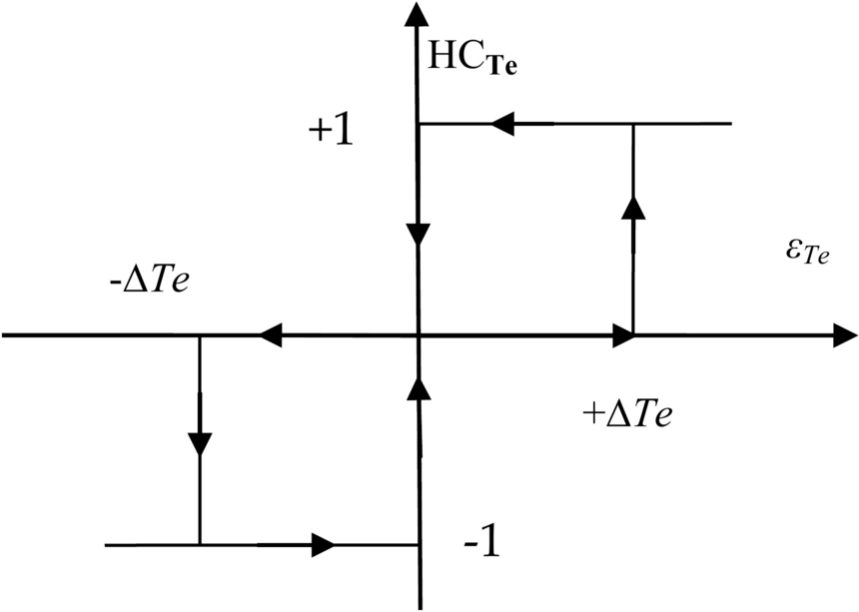
Torque HC regulator.

**Fig. 3:**
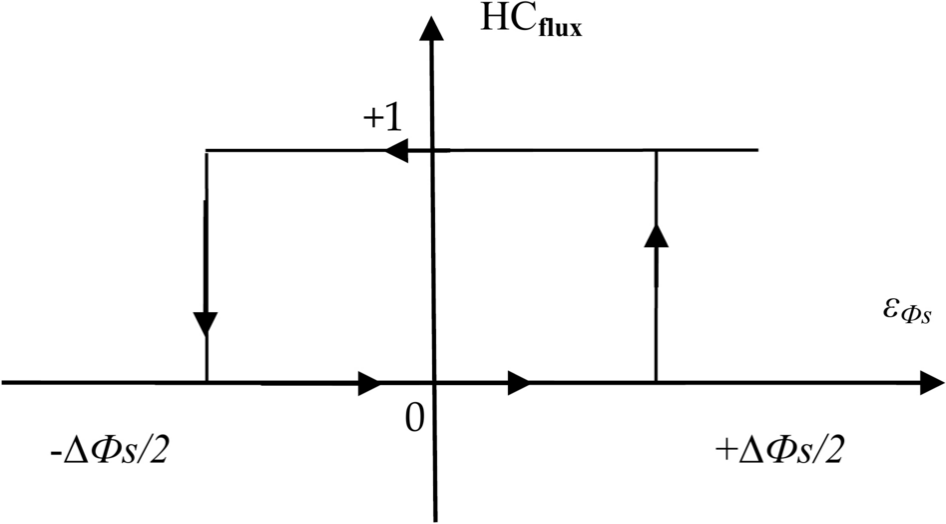
Flux HC regulator.

In this traditional DTC approach, 6 sectors 1 through 6 are used to control the flux beam. In each sector, only one voltage is applied, and this depends on the value of the output of the flux and torque regulator. For example, if the output of the torque regulator is 1 and the output of the flux regulator is 0, then voltage 4 is applied when sector 2 is, and voltage 6 is applied in sector 4. In Fig. 2 and Fig. 3, the shape of the regulators used in this approach is shown, where the torque regulator has three outputs: 0, -1, and 1. The outputs of the flux regulator are 0 and 1.

The relationship between voltage and flux is represented by the Eq. (8) ^44^.

8 $$\left\{\begin{array}{c}{V}_{s}={R}_{s}{i}_{s}+\frac{{d\Phi }_{s}}{dt}\\ {V}_{r}={R}_{r}{i}_{r}+\frac{{d\Phi }_{r}}{dt}\end{array}\right.$$

With: $${V}_{s}={V}_{s\alpha }+j{V}_{s\alpha }, {i}_{s}={i}_{s\alpha }+j{i}_{s\alpha }, {i}_{r}={i}_{r\alpha }+j{i}_{r\alpha }, {\Phi }_{s}={\Phi }_{s\alpha }+j{\Phi }_{s\alpha }, {\Phi }_{r}={\Phi }_{r\alpha }+j{\Phi }_{r\alpha }$$

Equation (8) can be written according to Eq. (9).

9 $$\frac{{d\Phi }_{s}}{dt}={V}_{s}-{R}_{s}{i}_{s}$$

Using Eq. (9), the flux can be written according to Eq. (10).

10 $${\Phi }_{s}={\int }_{0}^{t}({V}_{s}-{R}_{s}{i}_{s})dt$$

Equation (10) becomes as follows:

11 $${\Phi }_{s}={\Phi }_{s0}+{V}_{s}{T}_{e}-{R}_{s}{\int }_{0}^{t}({i}_{s})dt$$

where, ${\Phi }_{s0}$ is the flux value in *t* = 0 s.

The flux of the SCIM is related to the operation of the inverter, whereby using the operating pulses the flux can be calculated according to the following equation^45^:

12 $${\Phi }_{s}\left(t\right)=\frac{2}{3}{V}_{dc}\left({S}_{1}+a{S}_{2}+{a}^{2}{S}_{3}\right)+{\Phi }_{s0}-{R}_{s}{\int }_{0}^{t}({i}_{s})dt$$

So, stator flux modulus can be expressed by Eq. (13).

13 $$\left|{\Phi }_{s}\right|=\sqrt{({\Phi }_{s\alpha }^{2}+{\Phi }_{s\beta }^{2})}$$

Using Eq. (13), an angle for the flux can be calculated, and this angle is used to calculate the sectors needed to run the machine’s inverter.

14 $${\alpha }_{s}=arctg\frac{{\Phi }_{s\beta }}{{\Phi }_{s\alpha }}$$

The current of the SCIM can be expressed by the Eq. (15).

15 $${i}_{s}={i}_{\alpha }+j{i}_{\beta }$$

Equation (16) represents the current in the αβ axis^46^.

16 $$\left\{\begin{array}{c}{i}_{\alpha }=\sqrt{\frac{2}{3}} {i}_{s\alpha }\\ {i}_{s\beta }=\frac{1}{\sqrt{2}}({i}_{sb}-{i}_{sc})\end{array}\right.$$

Equation (17) can be used to estimate the torque of the SCIM. Therefore, to estimate the torque, the flux must be estimated first, as the torque estimate is related to both the flux and the current.

17 $${T}_{e}=\frac{3}{2}p({\Phi }_{s\alpha }.{I}_{s\beta }-{\Phi }_{s\beta }.{I}_{s\alpha })$$

According to the^26^, the DTC (6 sectors) technique is affected by the change in IM parameters, as it gives large torque fluctuations in the durability test. Also, an augment in the THD of the current is observed. These drawbacks can be reduced in the next section, where the use of NNs is proposed as a suitable solution to defeat these drawbacks.

## Six-sector neural DTC approach

**Fig. 4:**
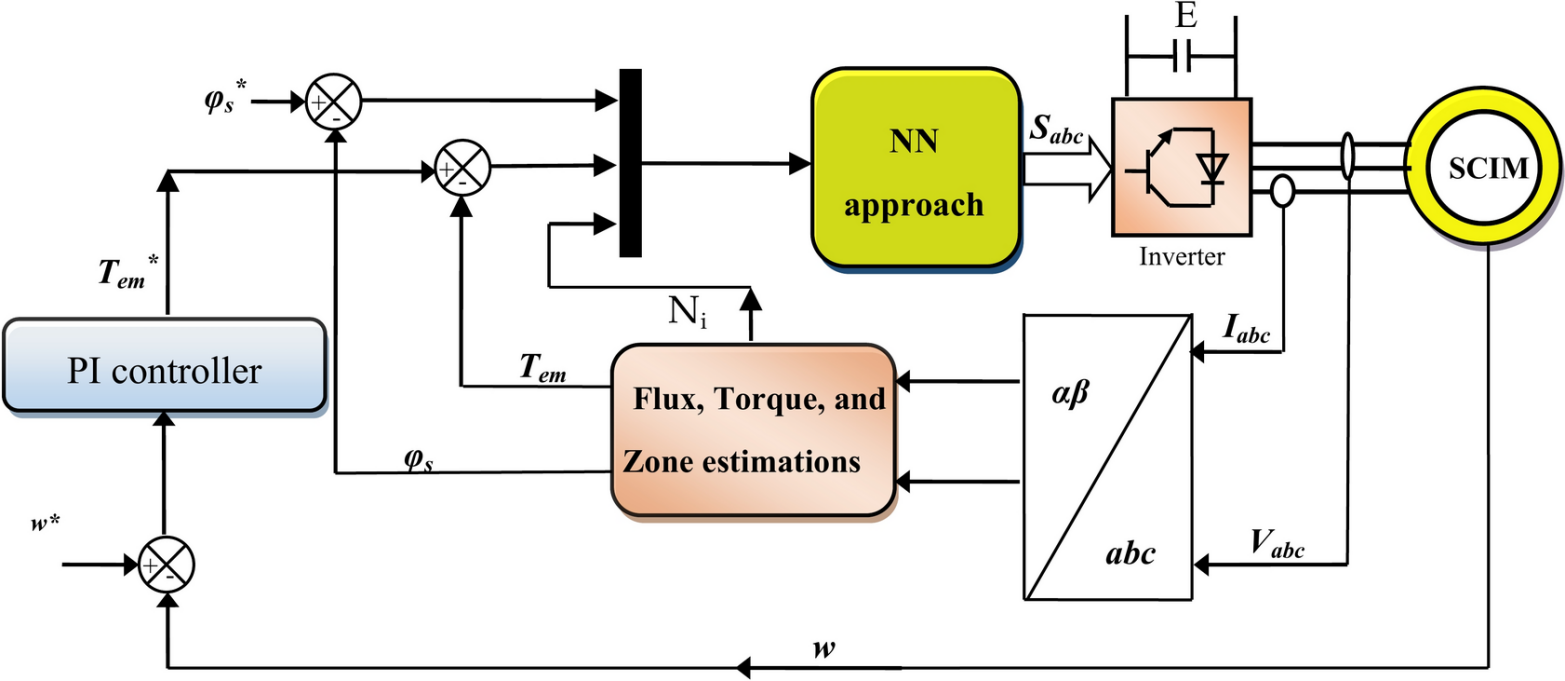
Six sectors neural DTC approach of SCIM.

In this section, an effective control is proposed that has the potential to overcome the problems of 6 sectors DTC technique. The use of NNs is the solution that was proposed in this section to compensate for both ST and HC in the 6 sectors DTC technique. Neural networks have been relied upon as a solution due to their accuracy and high robustness. Moreover, using the NN approach does not require knowing the exact MM of the studied system, as it only requires knowing the number of exits and the number of entrances. The NN strategy is characterized by a fast DR, which makes it one of the most appropriate and reliable solutions in the field of control. The designed control is described by its simplicity, inexpensive, ease of realization, fast DR, high robustness, and outstanding efficacy. This designed approach is a development of the 6 sectors DTC technique, as the same structure and simplicity that characterizes the 6 sectors DTC technique are maintained. Figure 4 represents the 6 sectors NN-DTC technique for SCIM control.

In the six-sector neural DTC approach, the traditional regulators are not used to command torque and flux, and the PWM technique is not used to control the machine’s inverter. In this approach, a single neural regulator is used to control torque and flux together. Also, using this regulator, the pulses necessary to run the SCIM inverter are produced. This designed approach does not require knowledge of the SCIM’s model, making it more robust and effective in handling changes in SCIM parameters.

The proposed approach has similarities with the 6 sectors DTC approach, as the same estimation equations found in the 6 sectors DTC technique are used. The proposed algorithm uses the PI regulator to determine the torque reference value, as the same regulator used in the 6 sectors DTC technique is used. The gain values ​​of this regulator are calculated using the simulation and experimentation method.

The neural controller used has three inputs and three outputs, where the outputs are the pulses to run the machine’s inverter. The characteristics of the neural regulator used are listed in Table 2.

**Table 2:** Characteristics of a used neural regulator.

Parameters	Values
Number of hidden layers	2
Number of neurons in the first hidden layer	64
Learning rate	0.01
Display step (error display interval)	25
Number of input layers	1
Number of iterations (epochs)	1000
Number of output layers	1
Number of neurons in input layer	3
Convergence acceleration coefficient (mc)	0.001
Number of neurons in output layer	3
Error (goal)	0.0001
Activation functions	Tansig and Purelin

**Fig. 5:**
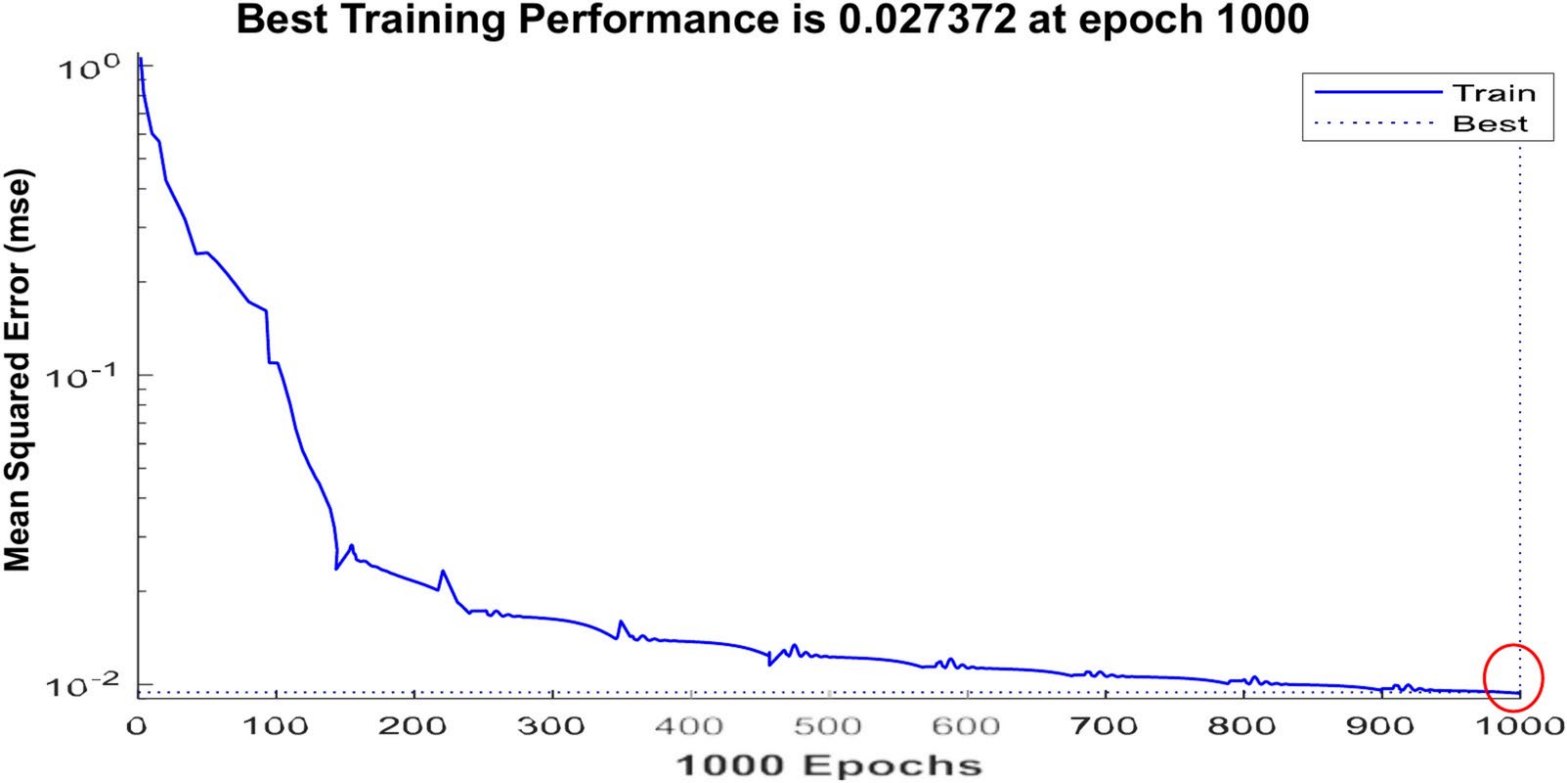
Training.

**Fig. 6:**
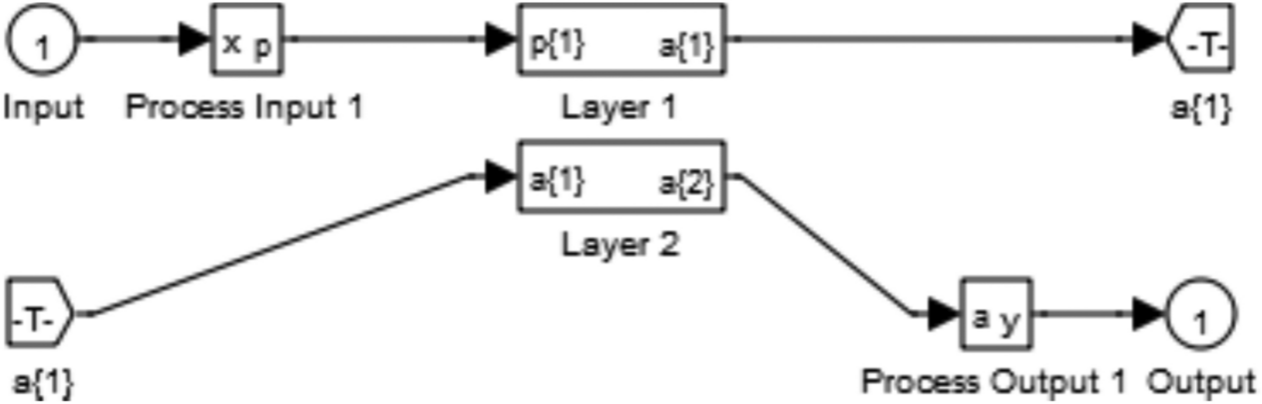
Neural controller from MATLAB.

**Fig. 7:**
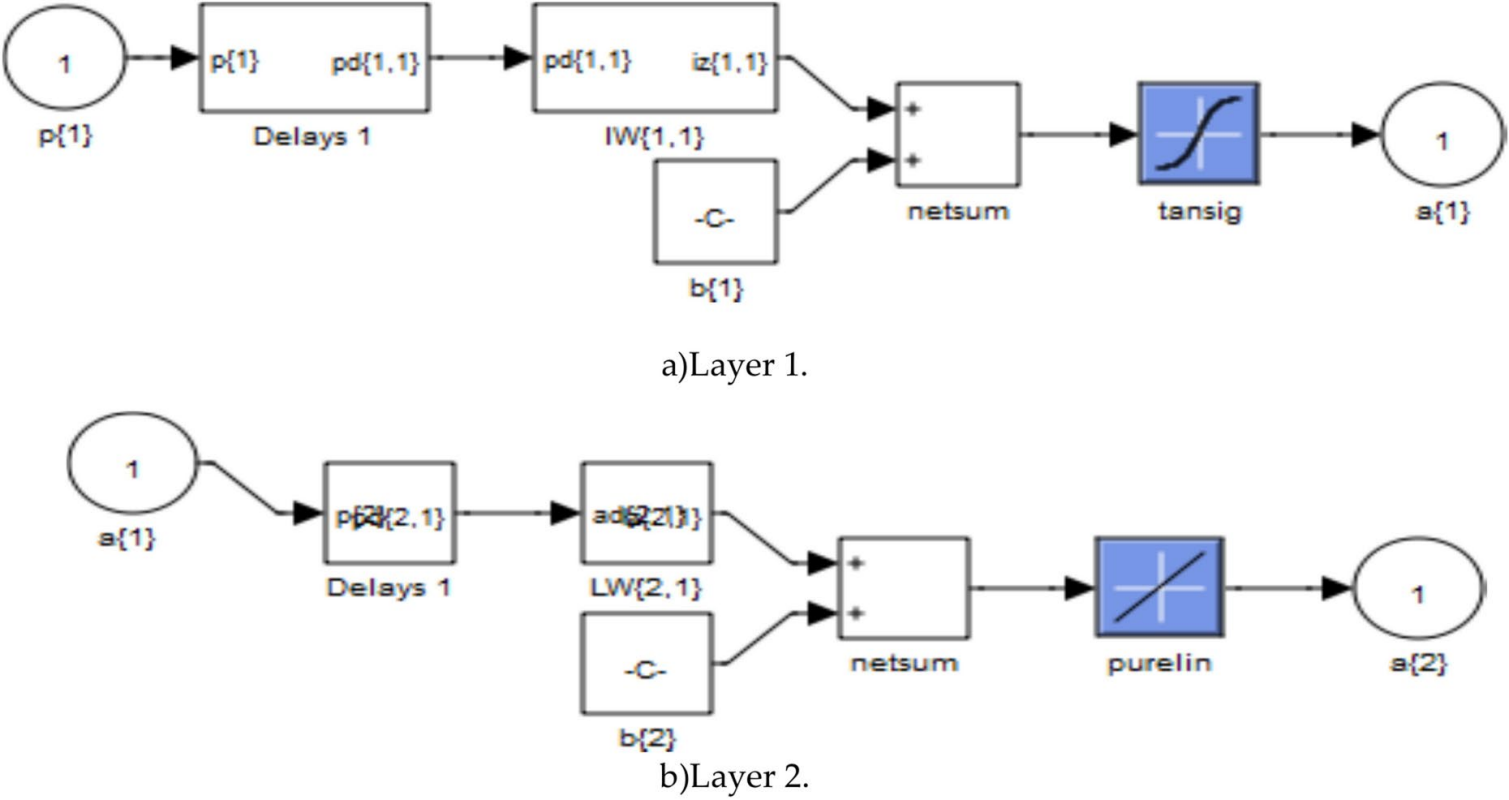
Structure of both layer 1 and 2.

Figure 5 represents the training of the neural controller used in this paper. Figures 6 and 7 represent the internal structure of the neural controller, layer 1, and layer 2.

Table 3 represents a comparison of the proposed approach with other existing strategies. In this table, the proposed approach is compared with DTC, BC, and FOC techniques, mentioning the similarities and differences. Also, they are compared in terms of performance and durability. The table is filled out based on the study of the works mentioned in the introduction section and the results obtained in the simulation section. The proposed approach has similarities with the DTC approach, as they use the same estimation equations for both torque and flux. Also, they are characterized by fast dynamic response and ease of implementation. Compared to the FOC approach, the proposed approach does not use a PI controller to control the characteristic magnitudes. Also, the PWM strategy is not used to generate the pulses necessary to operate the machine inverter. In the proposed approach, a neural controller is used to generate the impulses necessary to operate the inverter. The proposed approach has few gains which makes it easy to adjust compared to both BC and FOC techniques. Through the comparison made in Table 3, the proposed approach can be considered the best solution compared to DTC, FOC, and BC techniques.

**Table 3:** Compare the proposed approach with other strategies by mentioning the differences and similarities.

	**FOC**	**BC technique**	**DTC technique**	**Proposed technique**
Simplicity	No	No	Yes	Yes
Use the PWM or SVM strategy	Yes	Yes	No	No
Durability	Low	Medium	Low	High
Using a PI controller	Yes	No	No	No
Estimation of torque and flux	Yes	Yes	Yes	Yes
Hysteresis comparator	No	No	Yes	No
Value of torque ripples	High	Medium	High	Low
Ease of completion	Medium	High	Low	Low
THD value of current	High	Medium	High	Low
Dynamic response	Fast	Fast	Very fast	Very fast
Performance	Low	Medium	Medium	High
Switching table	No	No	Yes	No

In the next section, the 6-sector neural DTC approach is verified using simulation, where a 3.5 kW engine is used to test this approach.

## Results

To assess the competence of the designed approaches utilizing NNs and to conduct predictive analyses, a simulation model was established using MATLAB 2021a. The simulation of the 6-sector neural DTC approach for the Multi-Active Set (MAS) was carried out under the same conditions as those employed for the 6-sector DTC approach. A machine with the following parameters was used: *f* = 0.00325 Nm/rad/s, *J* = 0.043 kg·m^2^, *L*_*m*_ = 0.267 H, *L*_*s*_ = 0.336 H, *L*_*r*_ = 0.2036 H, *R*_*r*_ = 1.88 Ω, *R*_*s*_ = 4.28 Ω, *Ф*_*s*_ = 0.83 Wb, p = 2, *n*_*r*_ = 1480 rpm, *P*_*n*_ = 3.5 kW, *Te*_*n*_ = 20 N.m, *I*_*n*_ = 11.1/6.5 A, *V*_*n*_ = 230/400 V, and *f*_*s*_ = 50 Hz.

To identify the most effective control approach for the MAS driven by a two-level inverter, a comparative analysis between the two control methods previously examined (DTC and neural DTC) is imperative. Accordingly, we conducted the following experiments:

- No-load startup and steady-state conditions;
- Speed variation under no-load conditions;
- Application of resistive torque.

### Test 1: No-load startup and steady-state operation

**Fig. 8:**
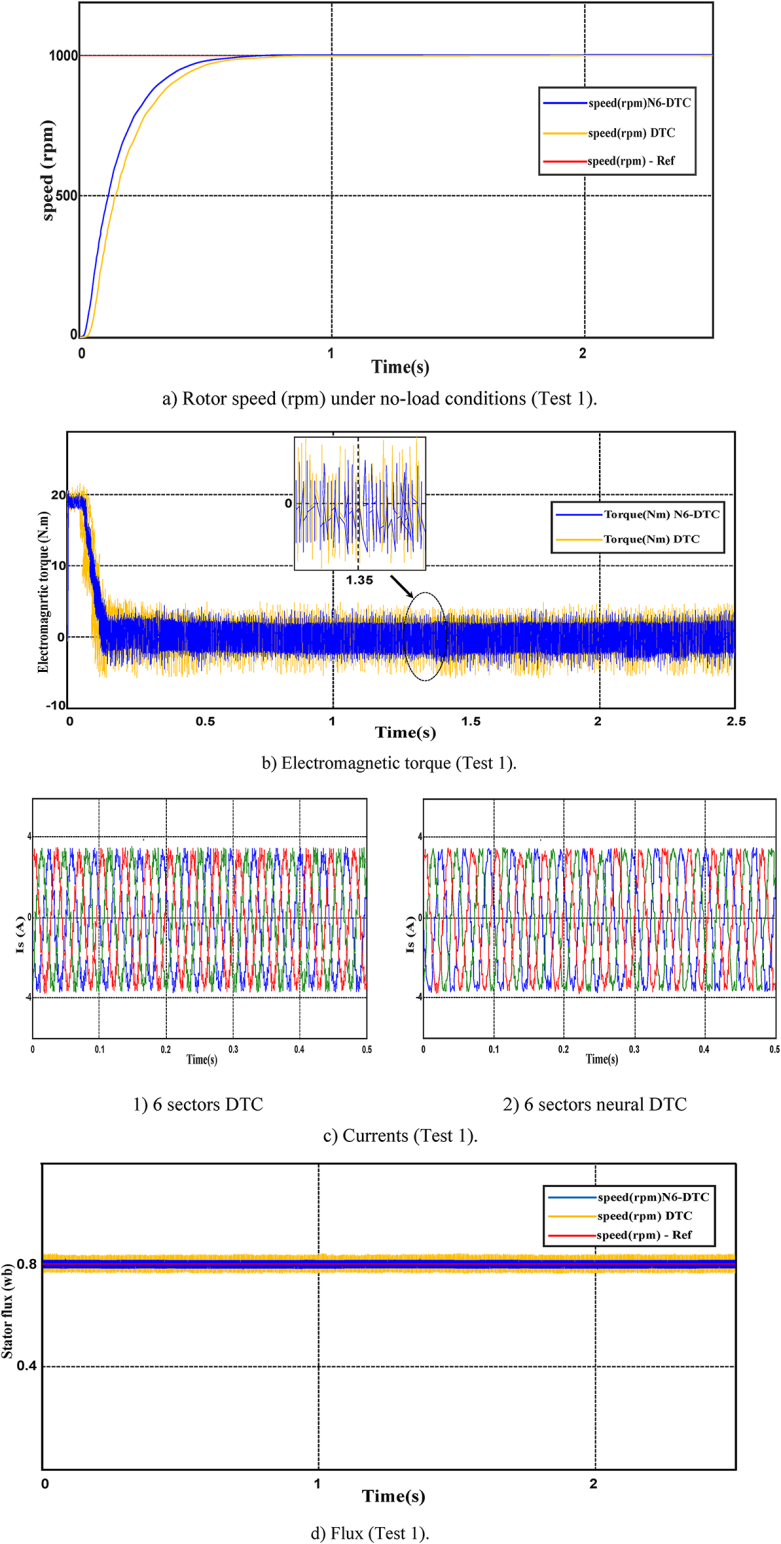

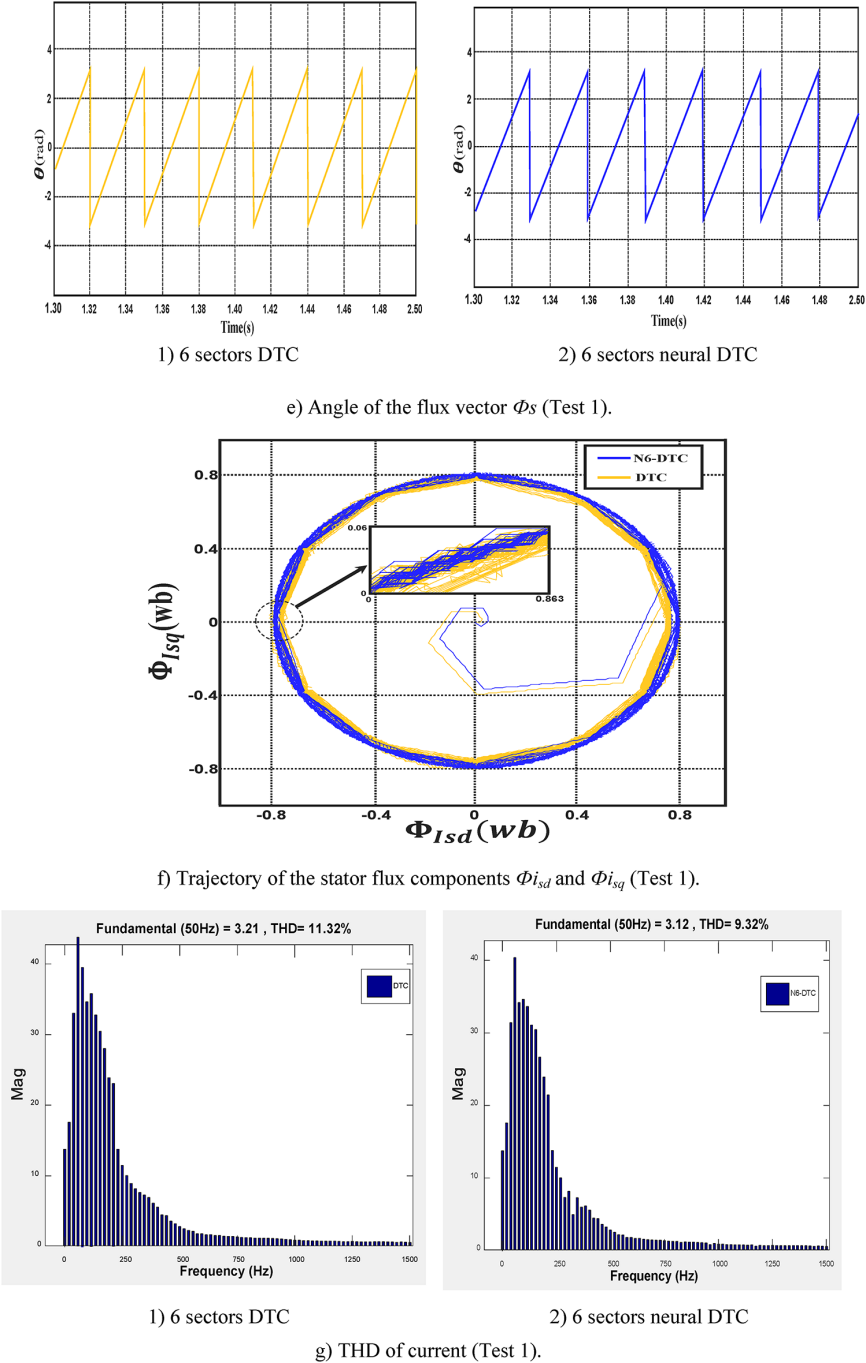
First test results.

In this test, the SCIM operates in the no-load condition with a speed reaching 1000 rpm. The results of this test are illustrated in Fig. 8.

Figure 8a illustrates the SCIM speed for both the DTC technique and 6 sectors of the neural DTC technique implemented in this study. The figure reveals that the speed displays a nonlinear transient response before achieving the predefined reference value. Importantly, the proposed control approach exhibits a more rapid response compared to the 6 sectors DTC technique. Furthermore, neither control algorithm results in overshooting the reference value. It is widely recognized that the response speed is closely associated with the control system architecture and the specific values of its parameters.

Figure 8b indicates that the torque reaches a maximum of approximately 18.7 N.m at the moment of SCIM activation. After this peak, the torque gradually declines and stabilizes, exhibiting noticeable ripple effects. Comparing the traditional DTC technique with the 6-sector neural DTC approach configuration reveals that the latter provides superior performance. The 6-sector neural DTC technique offers improved reference tracking and reduced ripple compared to the 6-sector DTC method.

Figure 8c represents the current for the two controls. This current takes a sinusoidal shape, noting that the quality of the current is higher if the 6 sectors neural DTC technique is used compared to the 6 sectors DTC technique.

Figure 8d represents the flux for the two approaches. The flux takes a constant value of 0.8 Wb with ripples. This flux has a fast DR to the two approaches. It is noted that the NN-DTC technique significantly reduced the flux ripples compared to the 6 sectors DTC technique.

Figure 8e represents the angle of the flux beam for the two approaches. It is noted that this angle has the same shape for the two approaches, as it takes the form of a saw tooth signal. Figure 8f represents the trajectory of the stator flux components *Фis*_*d*_ and *Фis*_*q*_ for the two algorithms. It is noted that the trajectory of this flux is circular for both approaches, with the 6-sector neural DTC technique having an advantage in terms of ripples compared to the 6-sector DTC technique. Figure 8g represents the THD value of a current of both algorithms. This value was 9.32% and 11.32% for both the 6 sectors’ neural DTC approach and the DTC approach, respectively. Therefore, the NN-DTC technique significantly reduced the THD value, as this reduction was estimated at 17.67%. This percentage shows the efficacy and efficiency of the NN-DTC technique in enhancing the quality of the current compared to the 6-sector DTC approach. The amplitude of the fundamental signal (50 Hz) was 3.21 A and 3.12 A for both the DTC approach and the proposed approach, respectively. Through these values, the proposed approach provided an unsatisfactory extension compared to the DTC technique. Therefore, the amplitude value can be considered negative in the proposed approach in this test. This negativity can be attributed to the gain values ​​of the proposed approach, which can be overcome in the future using the genetic algorithm or GWO technique.

### Test 2: Speed variation under no-load conditions

**Fig. 9:**
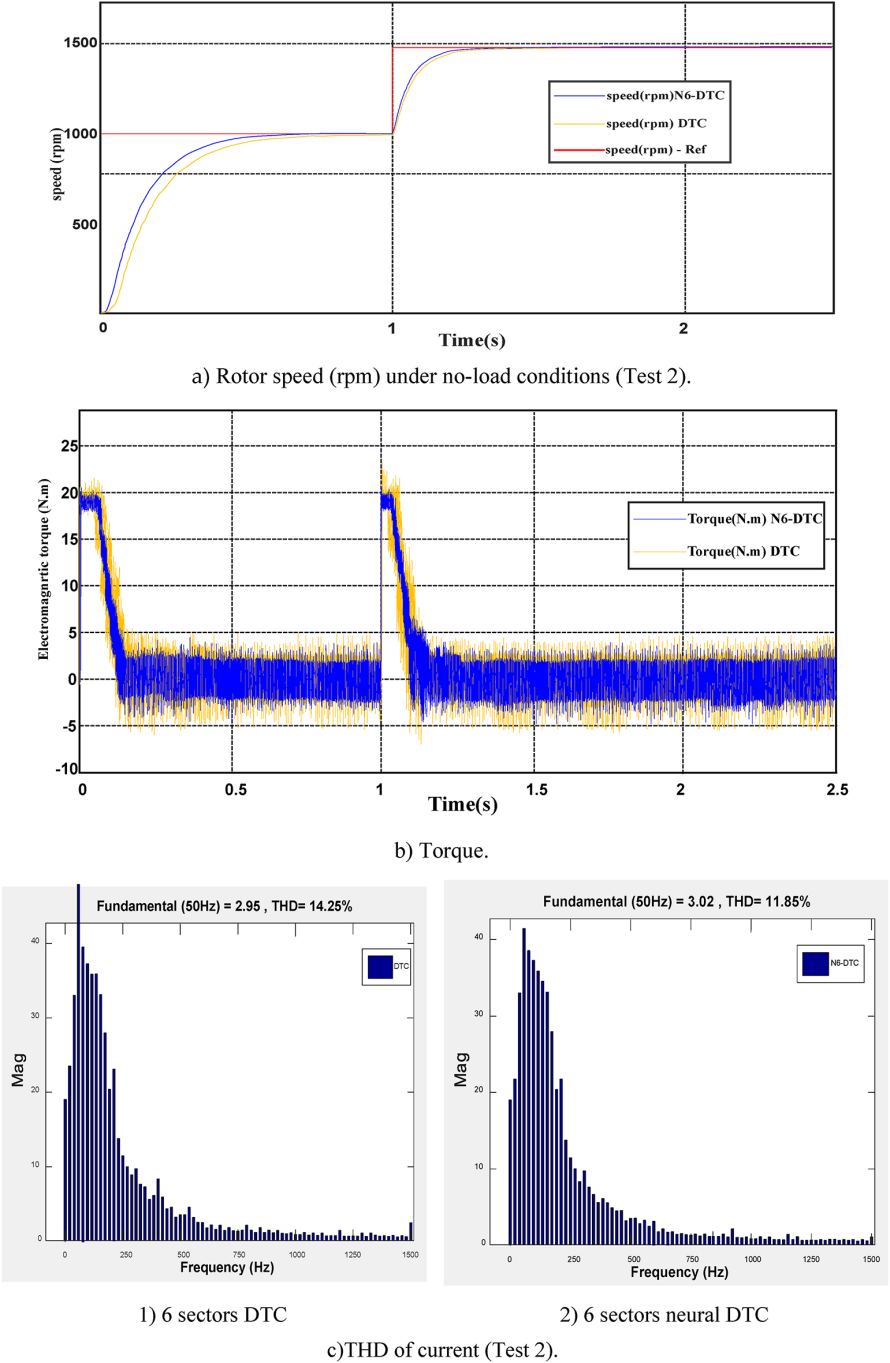
Second test results.

This test involves speed variation, where the SCIM speed transitions from 1000 to 1480 rpm at *t* = 1 s. Throughout the entire test, the SCIM operates under no-load conditions. The results of this evaluation are displayed in Fig. 9. Figure 9a represents the SCIM speed for the two algorithms. This speed follows the reference well for both algorithms, with the NN-DTC technique having an advantage in terms of response time compared to the DTC technique.

Figure 9b represents the torque for the two algorithms. This torque is affected by the change in speed at the moment of 1 s, where an augment in the value of the torque is observed and then it returns to zero. The torque returns to zero as a result of using the PI of the speed regulator. It is noted that the NN-DTC technique in this test minimized the torque undulations compared to the 6-sector DTC approach.

Figure 9c represents the THD value for the two algorithms. This value was 14.25% and 11.85% for both the DTC technique and the neural DTC technique, respectively. Therefore, the neural DTC technique minimized the THD value by an estimated 16.84%. This percentage indicates that the quality of the current in this test is high if the neural DTC technique is used. On the other hand, the amplitude value of the fundamental signal (50 Hz) in this test was 2.95 A and 3.02 A for both the DTC approach and the proposed approach, respectively. Through these values, the proposed approach provided greater scope than the DTC technique, which is a good thing that highlights the superiority of the proposed approach and its high performance. Therefore, the proposed approach improves the amplitude by an estimated rate of 2.32% compared to the DTC approach. This performance of the proposed approach makes it of future interest in other industrial applications.

Table 4 represents a study of the change in the value of both the THD value and the amplitude of the fundamental signal (50Hz) in the case of the first test and the second test. From Table 4, it is noted that the THD value increased in the second test compared to the first test for the two controls. Therefore, the THD value is affected by changing the rotation speed. The difference in THD value between the two tests was estimated at + 2.93% and + 2.53% for both the traditional DTC approach and the proposed approach. Through these values, it is noted that the proposed approach provided a lower difference to the THD value, which highlights the strength of the proposed approach compared to the traditional DTC approach. On the other hand, the amplitude value decreased in the second test compared to the first test for the two controls. This decrease is due to the change in speed in the second test compared to the first test. The difference in the amplitude value was estimated at 0.26 A and 0.10 A for both the traditional DTC strategy and the proposed approach, respectively. Through these values, it is noted that the proposed approach provided a smaller difference in amplitudes compared to the traditional DTC approach. Therefore, it can be said that the amplitude value was slightly affected if the proposed approach was used compared to the traditional DTC approach. These results highlight the effectiveness and robustness of the proposed approach compared to the traditional DTC approach.

**Table 4:** Effect study change of fundamental signal (50 Hz) amplitude and THD current in the second test compared to the first test.

		DTC approach	DTC-NN approach
Amplitude of fundamental signal (50 Hz)	**Test 1**	3.21	3.12
	**Test 2**	2.95	3.02
	**Test 2 – Test 1**	-0.26	-0.10
	**Ratios (%)**	-8.10	3.21
THD (%)	**Test 1**	11.32	9.32
	**Test 2**	14.25	11.85
	**Test 2 – Test 1**	+ 2.93	+ 2.53
	**Ratios (%)**	20.56	21.35

### Test with resistive torque (Load condition)

**Fig. 10:**
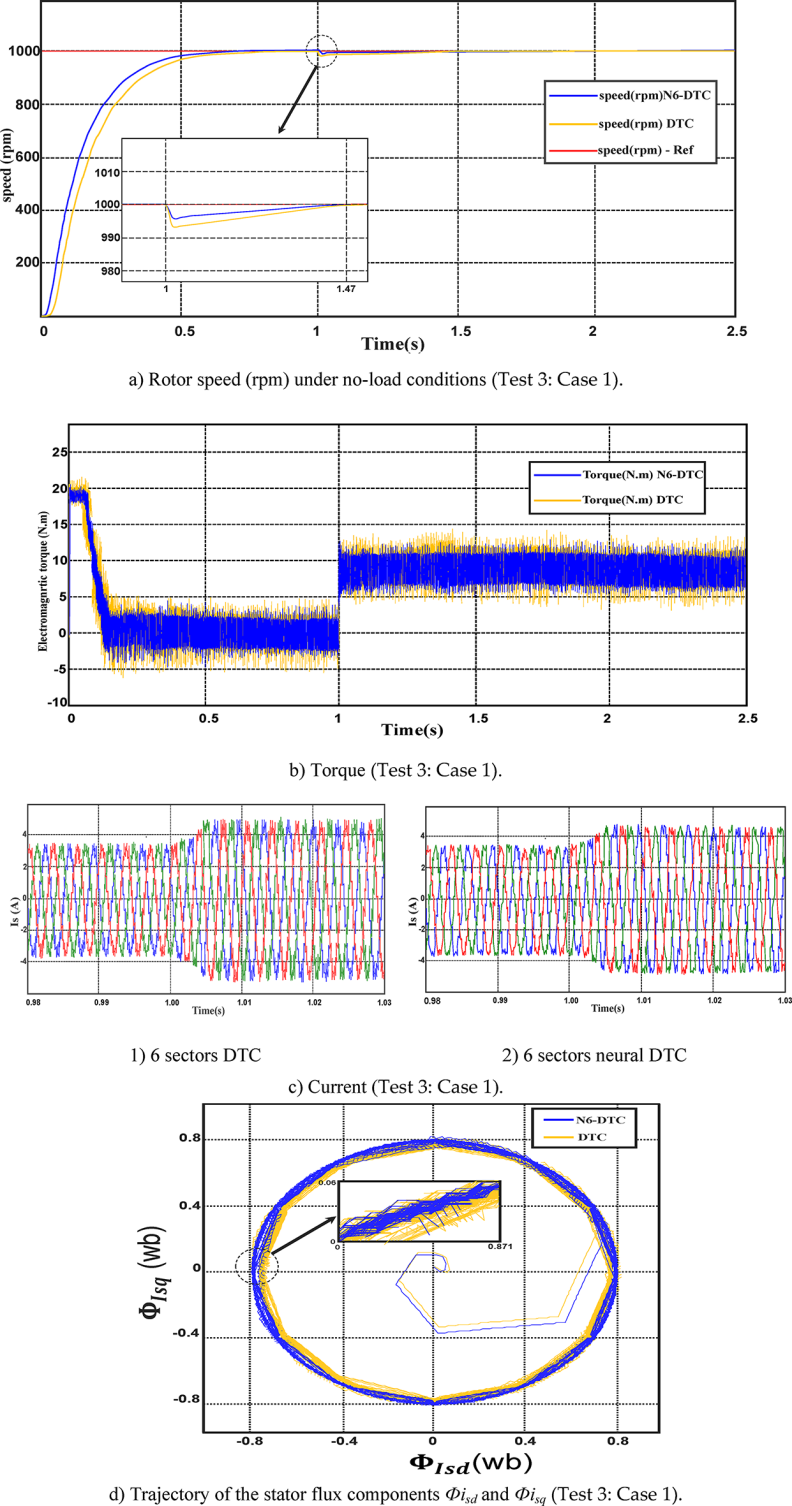
Third test results in the case 1.

**Fig. 11:**
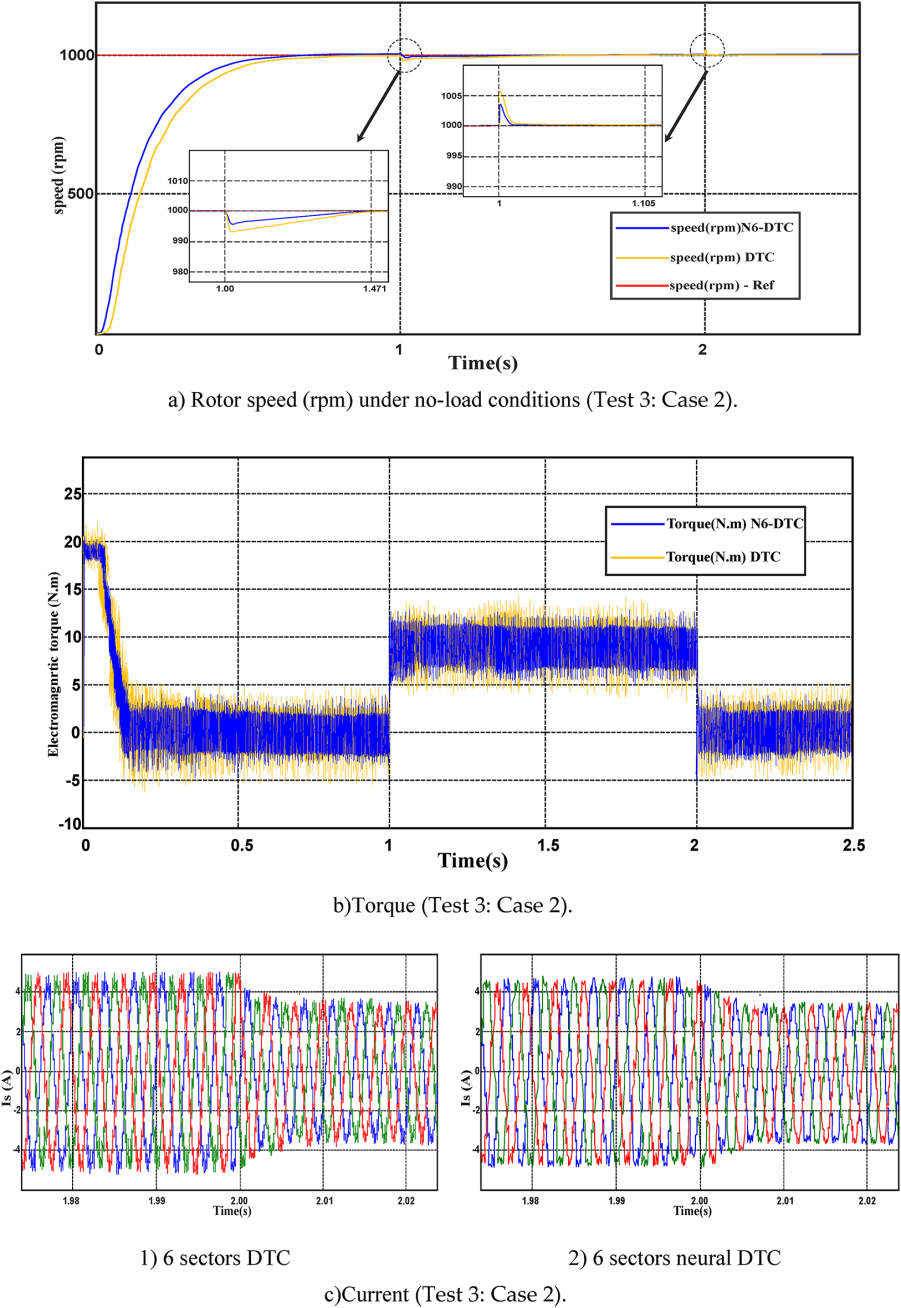
Third test results in the case 2.

In this test, the performance of the proposed 6-sector neural DTC approach was evaluated under load conditions while maintaining a constant speed. The obtained results were compared with those of the 6 sectors DTC approach and are depicted in Figs. 10 and 11.

**Case 1:** Apply a load in the time range of 1 to 2.5 s.

Figure 10a shows the speed response of both techniques, where a slight speed reduction is observed upon load application at t = 1 s with the proposed control. However, in both control methods, the speed remains close to the reference value due to the action of the PI regulator. Exceeding the limit value of speed at an instant of 1 s, its value was low in the case of using the 6 sectors neural DTC compared to the DTC technique, which highlights the effectiveness and efficiency of the NN-DTC technique in enhancing the features of the SCIM. Conversely, the torque response follows the applied load, with a rapid adjustment as the SCIM torque reaches 7 N.m (Fig. 10b). Additionally, torque ripples are evident in both cases, but the proposed 6-sector neural DTC approach exhibits significantly reduced ripple compared to the 6-sector DTC technique.

Figure 10c represents the current for the two algorithms. This current has a sinusoidal shape, with the NN-DTC technique having an advantage in terms of quality compared to the DTC technique. It is noted that the value of the current increased at the time point of 1 s as a result of applying a load, which is normal because the current is related to torque.

Figure 10d represents the trajectory of the stator flux components *Фi*_*sd*_ and *Фi*_*sq*_ of both algorithms. From this figure, it is noted that the trajectory takes a circular shape for the two algorithms, with the diameter of this circle being 1.6 Wb, with the NN-DTC technique having an advantage in terms of ripples compared to the DTC technique.

**Case 2:** Apply a load in the time range 1 to 2 s.

In Fig. 11, the SCIM is tested if the load is applied in the time range from 1 to 2 s. In this test, the load is removed after 2 s and the speed is fixed at 1000 rpm. From this figure, it is noted that the speed follows the reference well with an exceedance of the limit value at the time points of 1 and 2 s (Fig. 11a). The value of this override is lower if the neural DTC technique is used compared to the DTC technique. Also, it is noted that the 6-sector neural DTC technique gave a better speed response time than the DTC technique. The torque of the SCIM of the two approaches used is represented in Fig. 11b. The torques follows the reference well, with an advantage to the neural DTC technique in terms of undulations and overshoots value compared to the DTC technique.

The SCIM current in this second case is represented in Fig. 11c, where this current remains sinusoidal in shape with larger ripples in the case of using the 6 sectors DTC technique compared to the neural DTC technique. It is noted that the value of the current at the time point of 2 s has decreased significantly and this is a result of the load being removed at this moment. This decrease is normal because the current increases when the load is applied and decreases when it is removed.

## Experimental results

In this section, the experimental work carried out is divided into three main parts:

- Power section
- Control section
- Measurement environment

**Fig. 12:**
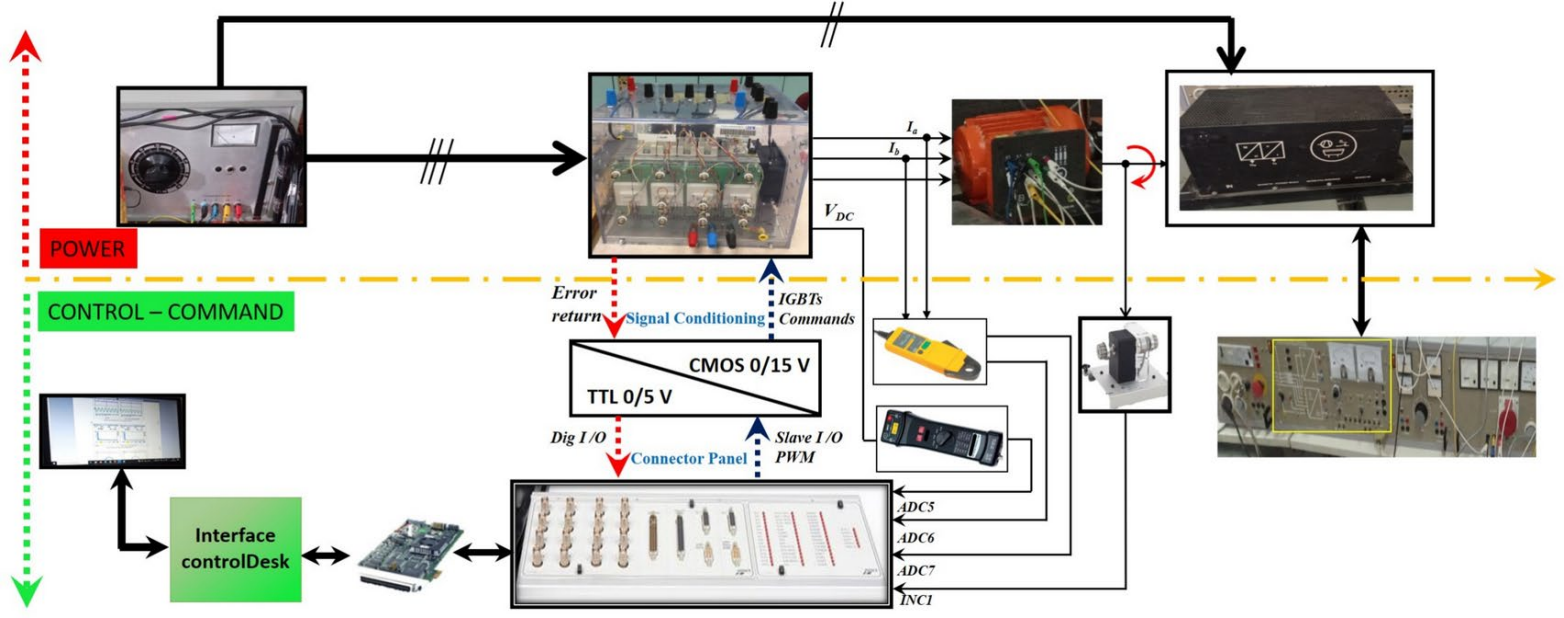
Schematic diagram of the experimental platform.

In this experimental work, the same SCIM used in the simulation is used. The load applied to SCIM in this experimental work is a synchronous motor (SM). Figure 12 presents a schematic diagram of the experimental platform used in this project.

### Power section

The “POWER” part consists of an IGBT voltage inverter and two machines: a 3.5 kW SCIM with squirrel cage and a 5.5 kW SM. These are coupled and the SM is used as the load of the SCIM.

### Control–command section

The “Control” section revolves around the DS1104 R&D Controller Board developed by the German company dSPACE, which is housed in a computer. This control board consists of two processors. The master processor generates PWM control signals in TTL 0/5 V logic, forming the hardware component of the dSPACE system.

The software component comprises two key software tools. The first, MATLAB 2021a, facilitates easy programming of real-time applications in Simulink by using specific blocks from the Real-Time Interface (RTI) toolbox, which allows configuration of the DS1104 board’s inputs and outputs. The second software, Control Desk, is used to load the program code onto the board (written graphically in Simulink, compiled, and transformed into C code), to create a complete experimental environment, including a graphical interface for real-time process control, data processing, and storage in a format compatible with MATLAB 2021a for further analysis. It also enables real-time monitoring of measured or calculated data using graphical or digital displays.

The master processor manages the application, while the slave processor, a TEXAS INSTRUMENT DSP (Digital Signal Processor) type TMS320F240, handles additional processing tasks.

### Measurement environment

Information exchange between the aforementioned components is managed via an external connector panel (CP1104 /dSPACE), linked to the board through a shielded cable. This panel facilitates the reception of analog signals through BNC connectors, signal conditioning for PWM control signals, and potential error signals from the Semikron converter, and integrates various measurement sensors.

The signal conditioning interface performs conversion between TTL 0/5 V logic and CMOS 0/15 V logic. This conversion is crucial as the DS1104 control board operates with TTL 0/5 V signals, while the inverter requires CMOS 0/15 V signals.

The measurement environment includes LEM LA55TP closed-loop Hall effect current sensors for current measurement, LEM LV100-500 closed-loop Hall effect voltage sensors for voltage measurement, and an incremental encoder for measuring SCIM speed. To ensure accurate digital processing, the analog currents must be sampled. To prevent aliasing, a guard filter with a cut-off frequency of approximately 500 Hz (significantly higher than the fundamental frequency of 50 Hz) is employed between each sensor and the analog-to-digital converter.

**Fig. 13:**
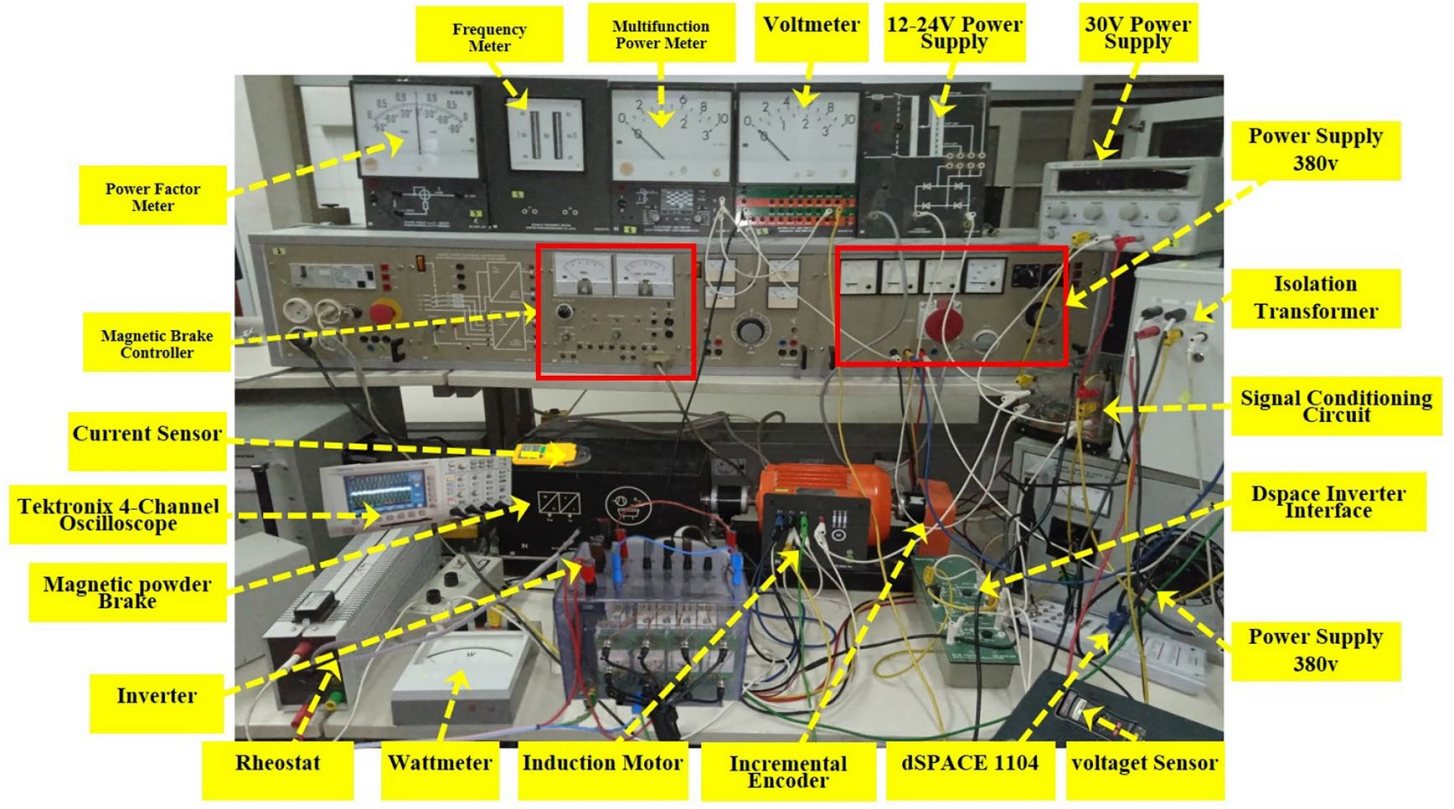
Experimental platform.

Figure 13 represents the real picture of the experimental work done in this paper. From this figure, it is possible to identify all the necessary equipment used to carry out this experimental work.

**Fig. 14:**
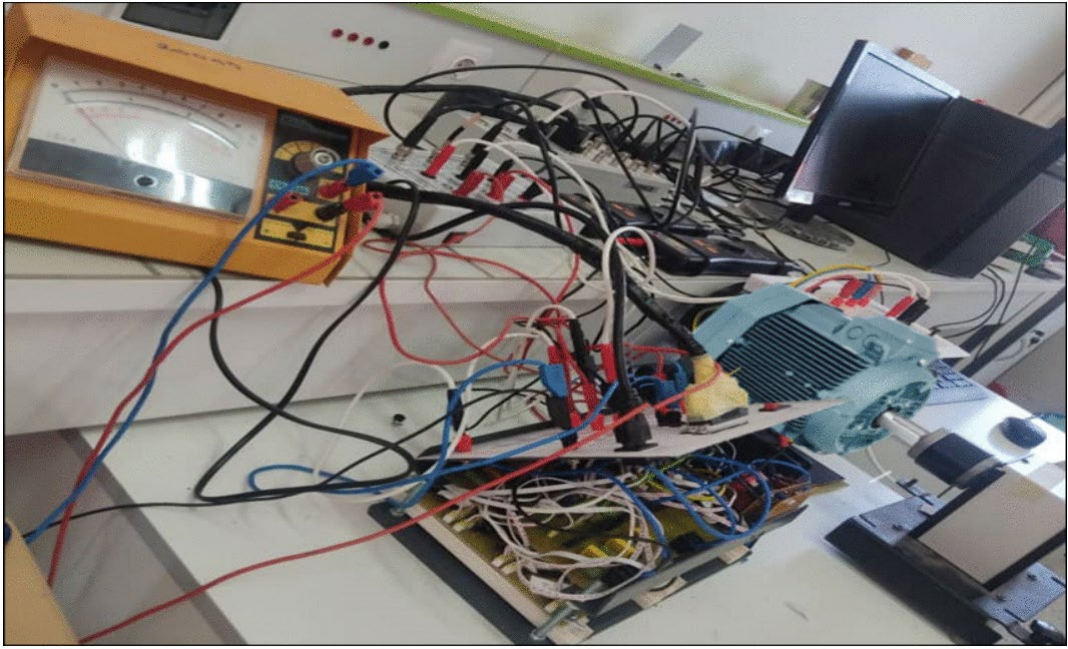
Experimental platform.

Figure 14 represents another picture of the experimental work, where the measurement performed (voltage measurement) on one of the components of the studied control system is illustrated.

Three different tests were performed on the machine, as follows:

### Without speed

**Fig. 15:**
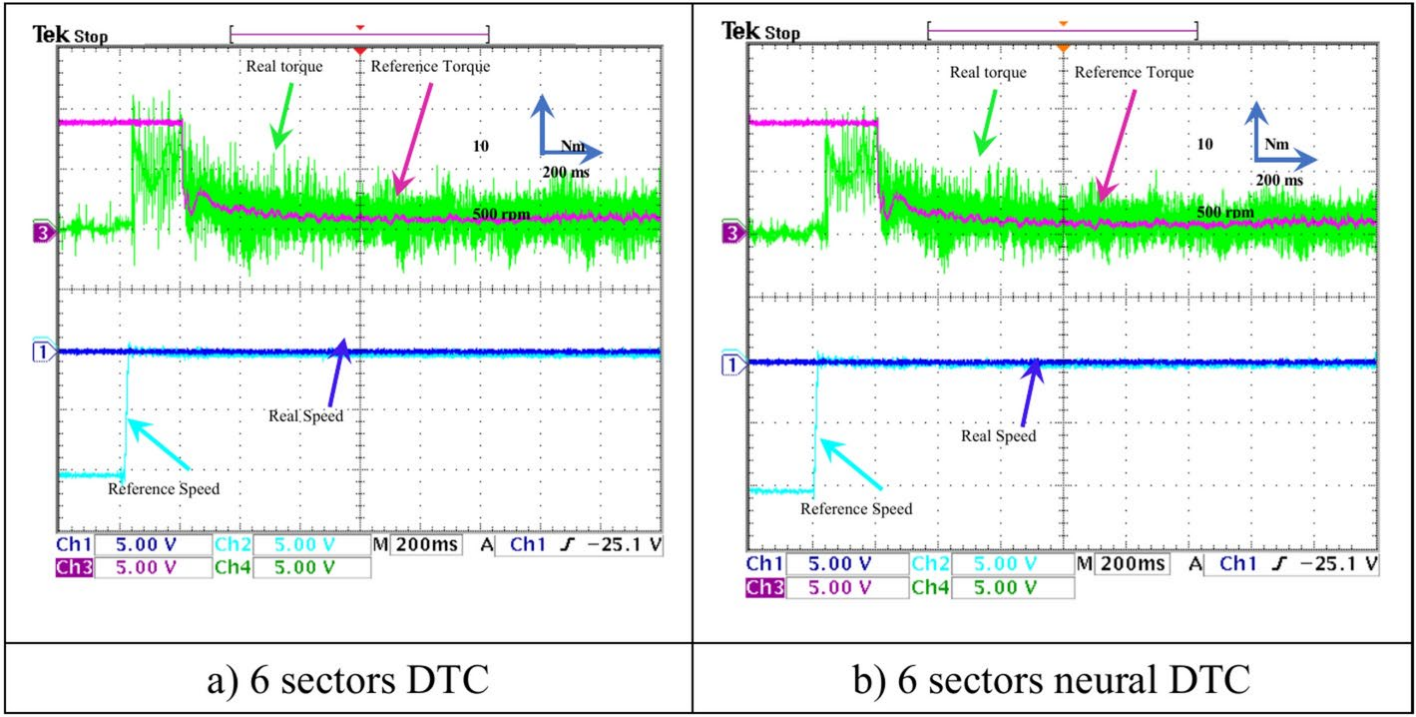
Torque and speed.

**Fig. 16:**
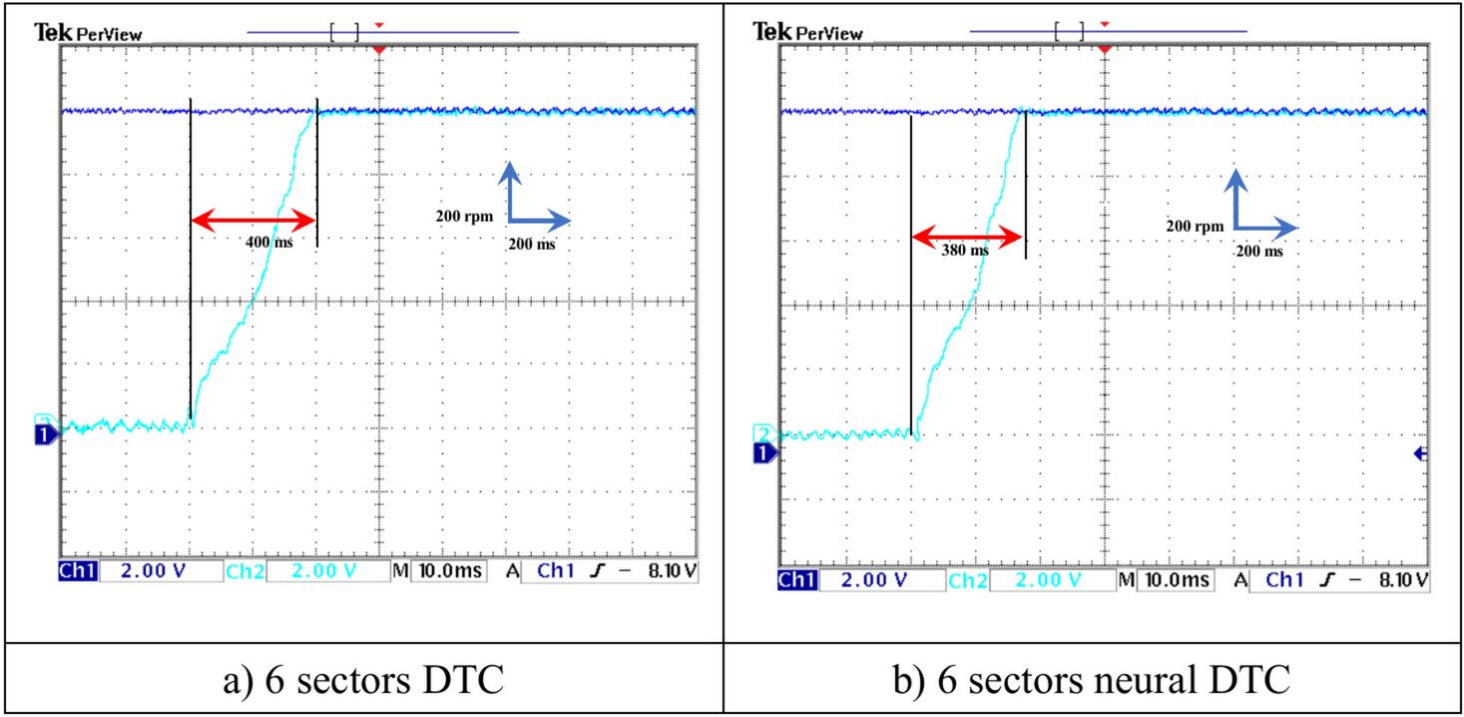
Zoom rotor speed (no-load and without speed variation).

**Fig. 17:**
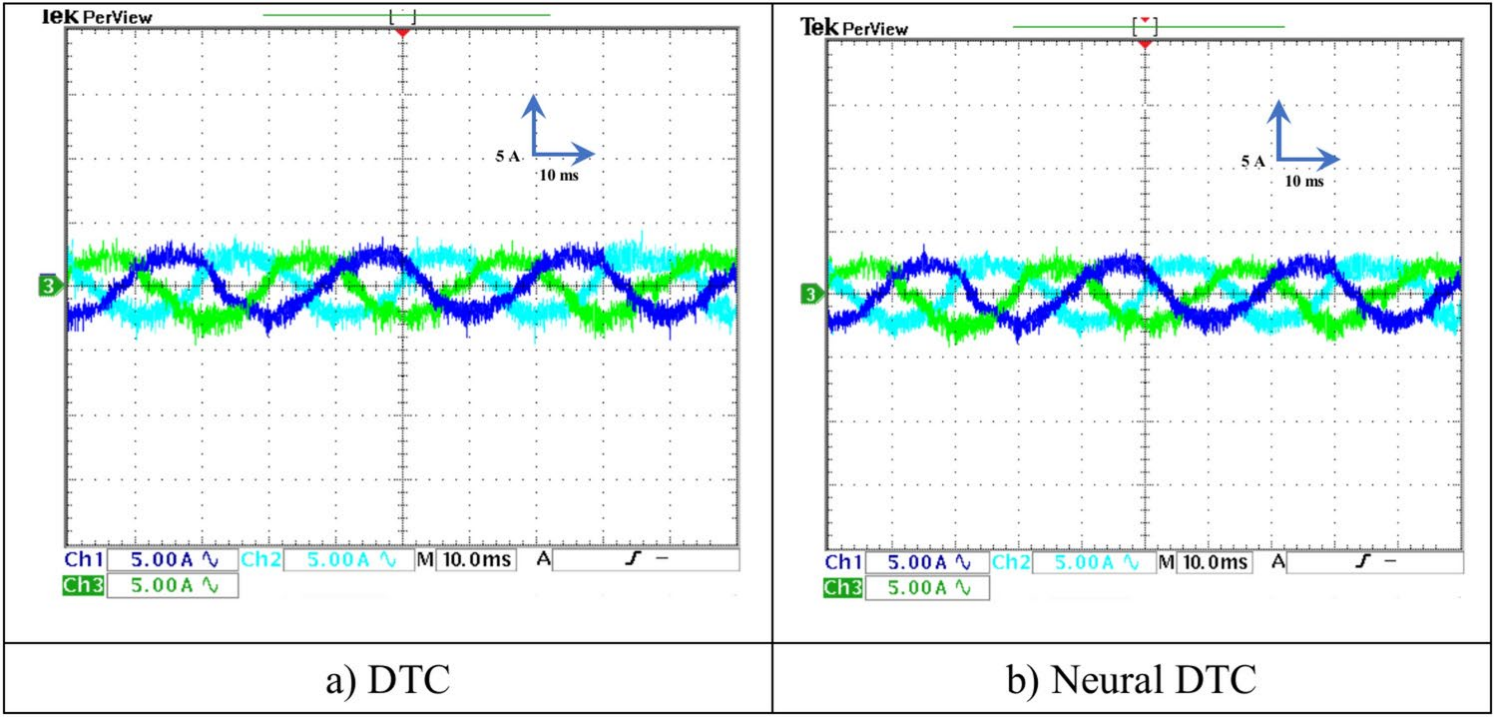
Zoom in the steady-state regime of the stator currents (no-load and without speed variation).

**Fig. 18:**
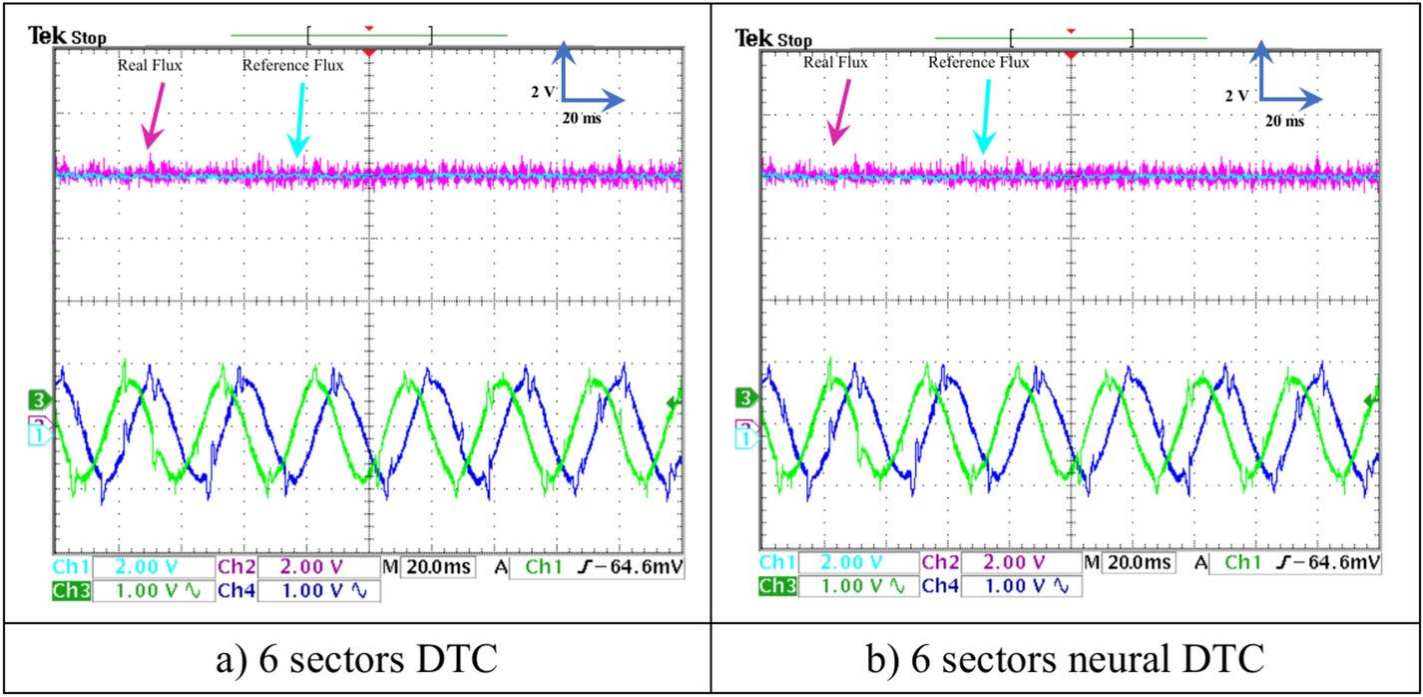
Stator flux and their components (when no load is applied without change in speed).

In this test, a torque meter was used: 5 V and a gain of 0.1; for the flux, a gain of 1 and a scale of 2 V. Also, the SCIM speed is fixed at a value of 1000 RPM while performing the necessary measurements of current, flux, speed, and torque for the control methods proposed in this work. Figure 15 shows the experimental results for torque and speed in the case of using the DTC technique and neural DTC technique. The torque and speed follow the references well, with an advantage over the neural DTC technique in terms of ripples and response time, which are the same results obtained in simulation. The proposed approach has a fast DR compared to the 6 sectors DTC technique, and this is shown in Fig. 16, where it is noted that the speed response time was 400 ms and 380 ms for both the DTC technique and the neural DTC technique, respectively. These experimental results demonstrate the performance obtained in the simulation and demonstrate the efficiency and competence of the 6 sectors’ neural DTC approach in improving the speed response time value. Figure 17 shows the zoom of stator currents (1 div = 5A) absorbed by the SCIM, where these results are experimental for the three control techniques. It can be seen that the neural DTC technique shows a better sinusoidal waveform of the current. Figure 18 shows the flux and its components for both control methods, where the flux takes a constant value and follows the reference value well with fluctuations (1 div = 0.2 Wb). The 6 sectors neural DTC technique exhibit very low ripples compared to the 6 sectors DTC technique. Additionally, the stator flux components take a sinusoidal shape in the case of the neural DTC technique compared to the DTC technique.

**Fig. 19:**
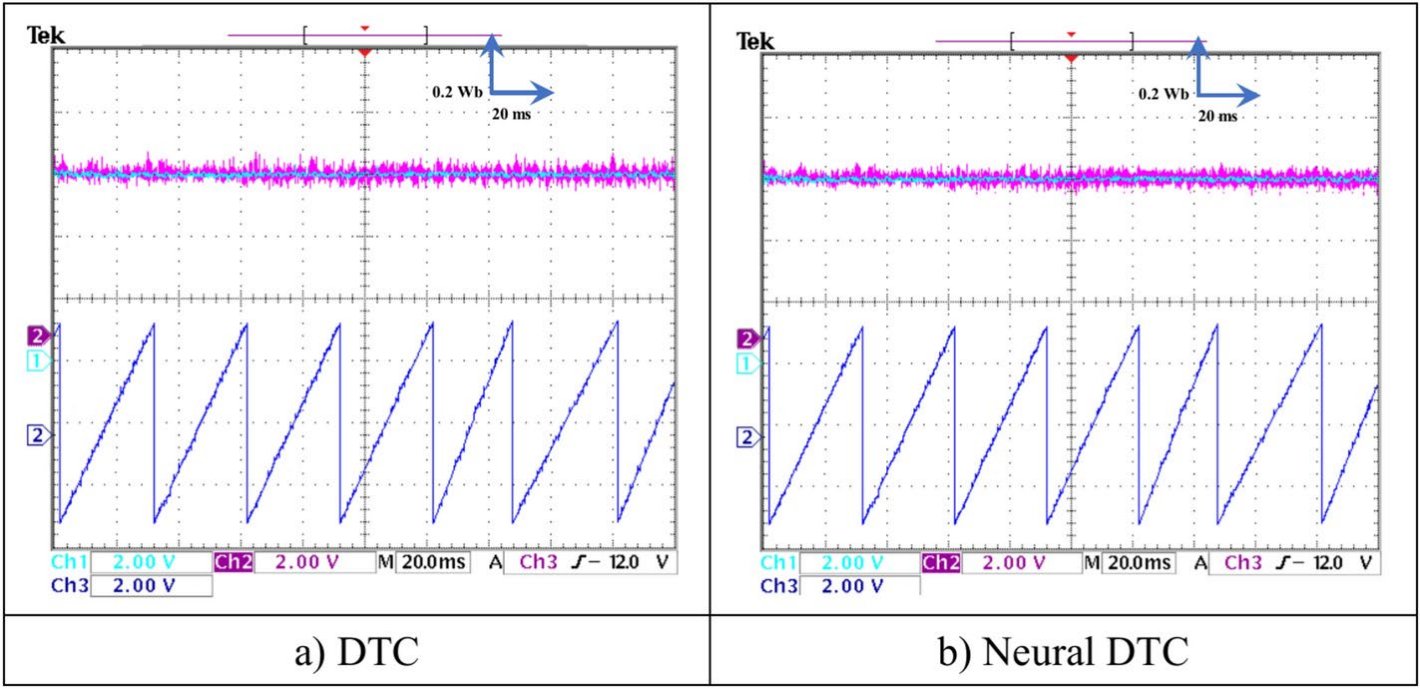
Stator flux and position (no-load and without speed variation).

**Fig. 20:**
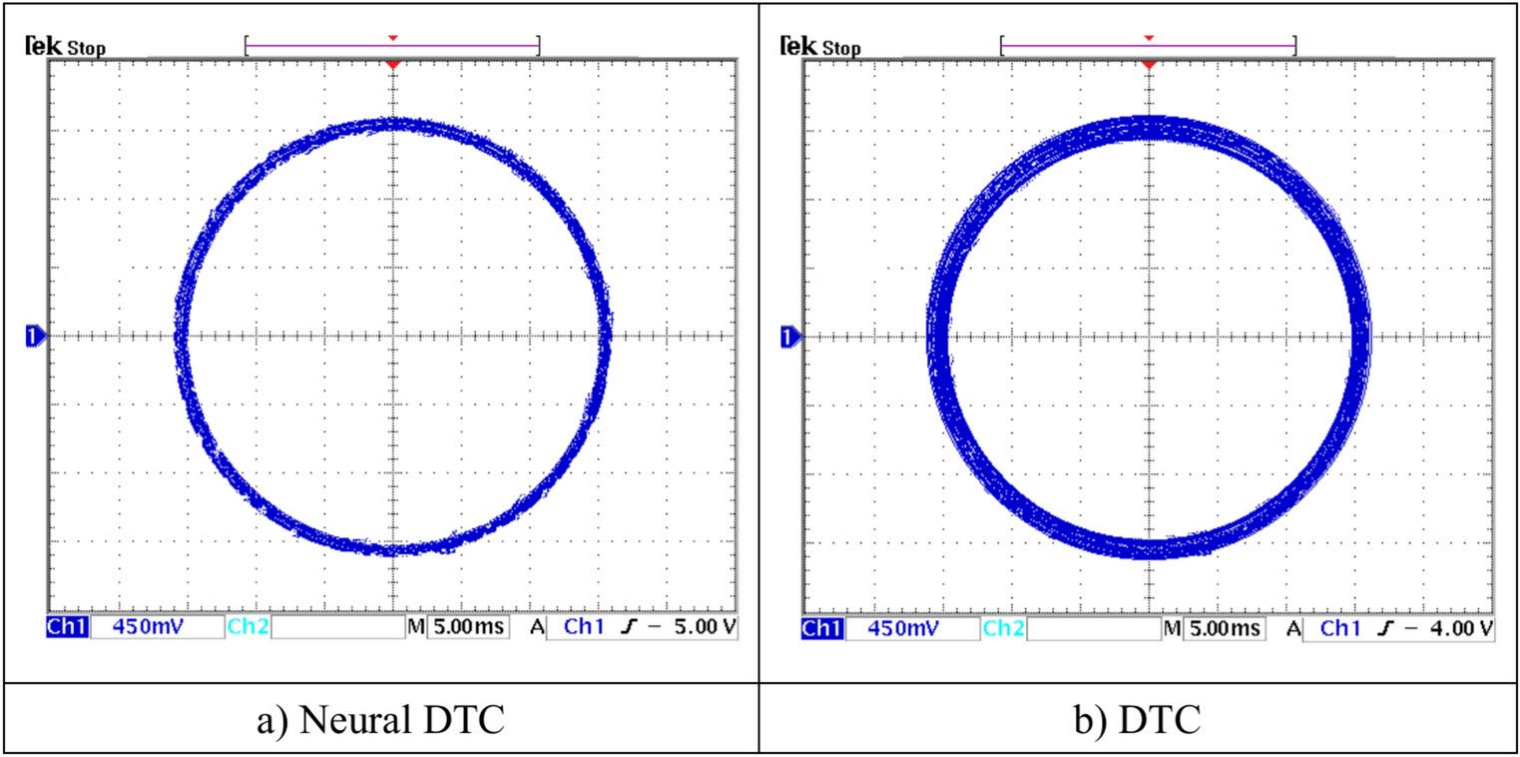
Evolution of stator flux components.

In Fig. 19, the zoom is given for the stator flux (1 div = 0.2 Wb) and the position for the two approaches. Additionally, the theta angle takes the form of a sawtooth signal, which is the same as that of the simulation. Figure 20 represents the evolution of flux stability obtained experimentally for the two controls. It is noted that the shape of the evolution of the flux components is a circular shape for the two controls, with an advantage for the neural DTC technique compared to the DTC technique in terms of ripples, and these are the same results obtained using simulation.

**Fig. 21:**
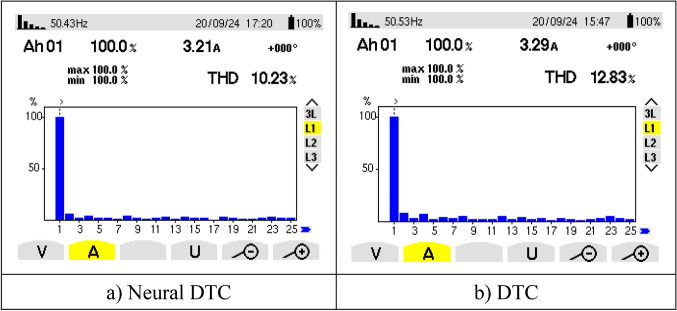
THD of current (First experimental test).

Figure 21 represents the value of THD and amplitude of a fundamental signal (50 Hz) for two controls. From this figure, it is noted that the amplitude and THD values ​​for the traditional DTC approach were estimated at 3.29 A and 12.83%, respectively. In the case of the proposed approach, the amplitude and THD values ​​were estimated at 3.21 A and 10.23%, respectively. Through these values, the proposed approach reduces the values ​​of both amplitude and THD by rates estimated at 2.43% and 20.27%, respectively. The amplitude value in this test can be considered negative for the proposed approach. This negativity can be attributed to the choice of characteristics of the NN technique used. As is known, NNs depend on experience and experimentation in choosing the number of internal layers and the number of epochs. In the future, this negativity can be overcome by using other strategies.

### With speed variation

**Fig. 22:**
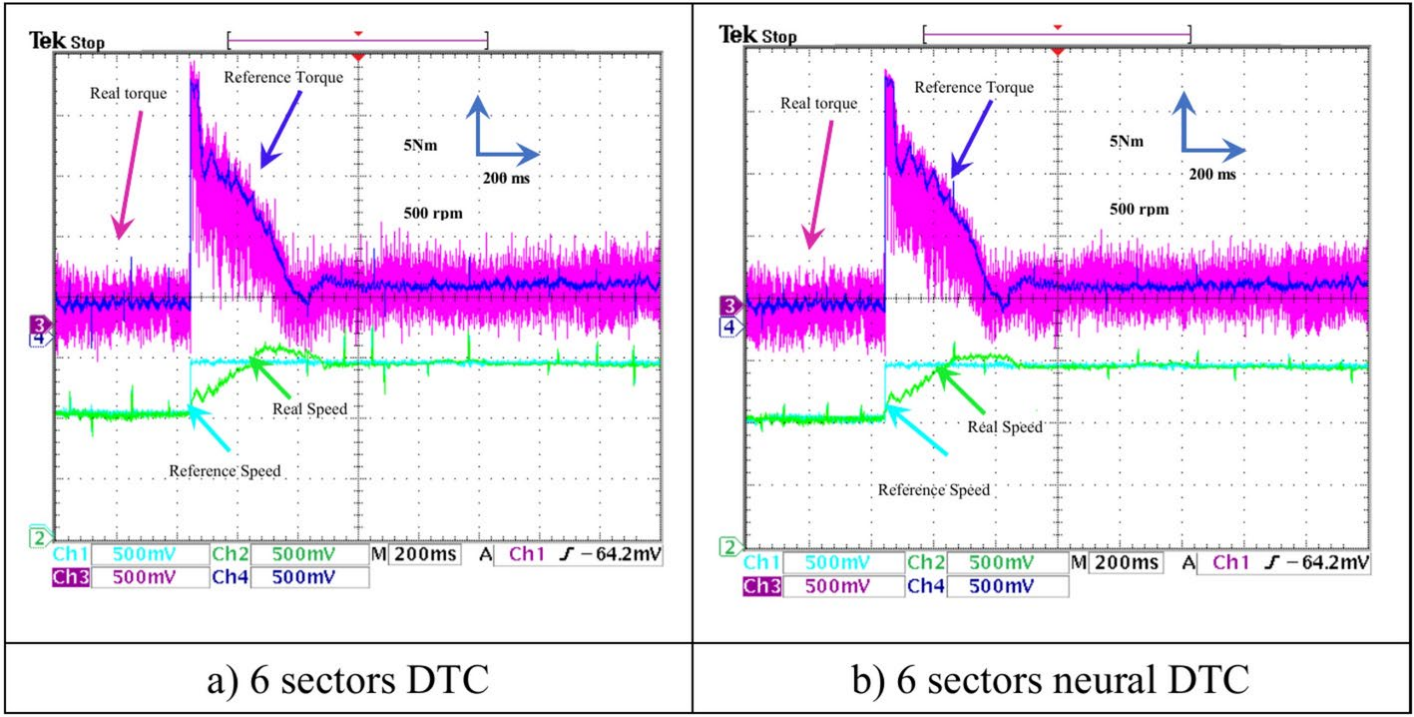
Torque and rotor speed (no-load and with speed variation).

**Fig. 23:**
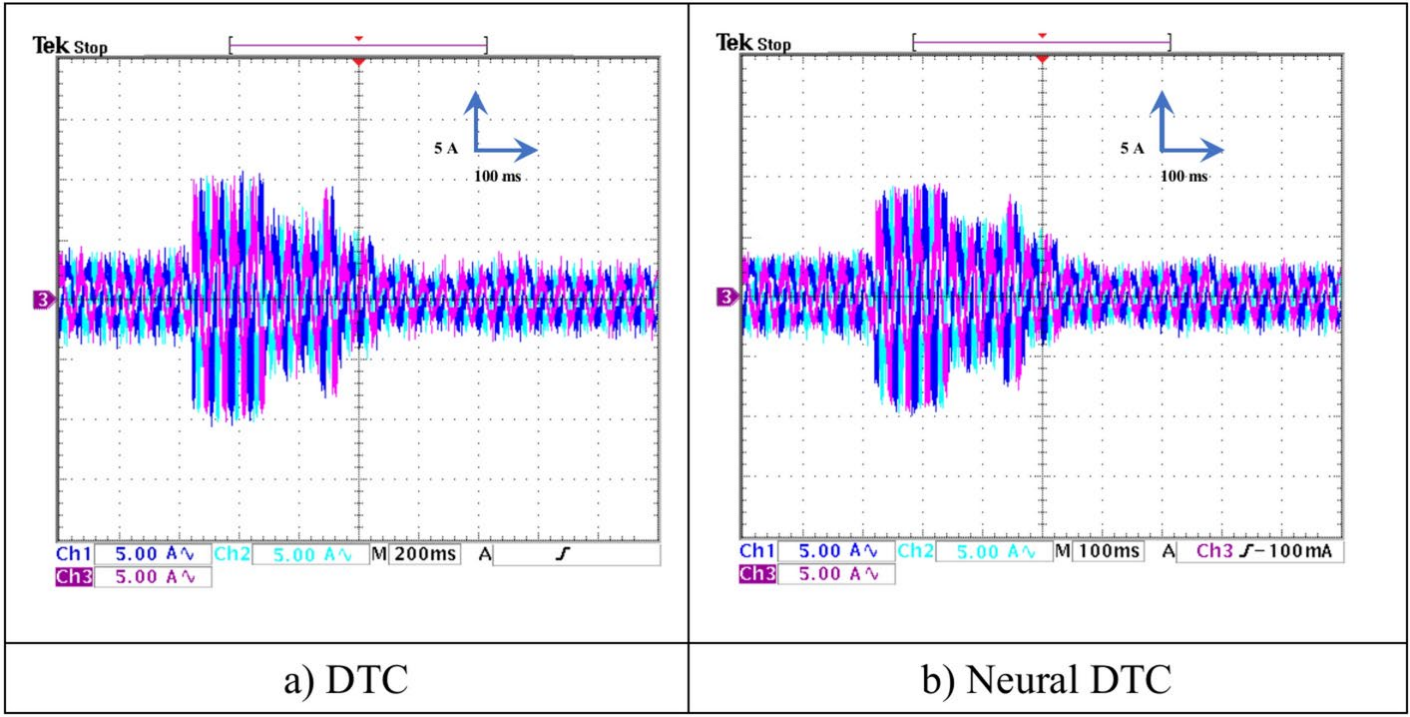
Stator currents (no-load and with speed variation).

**Fig. 24:**
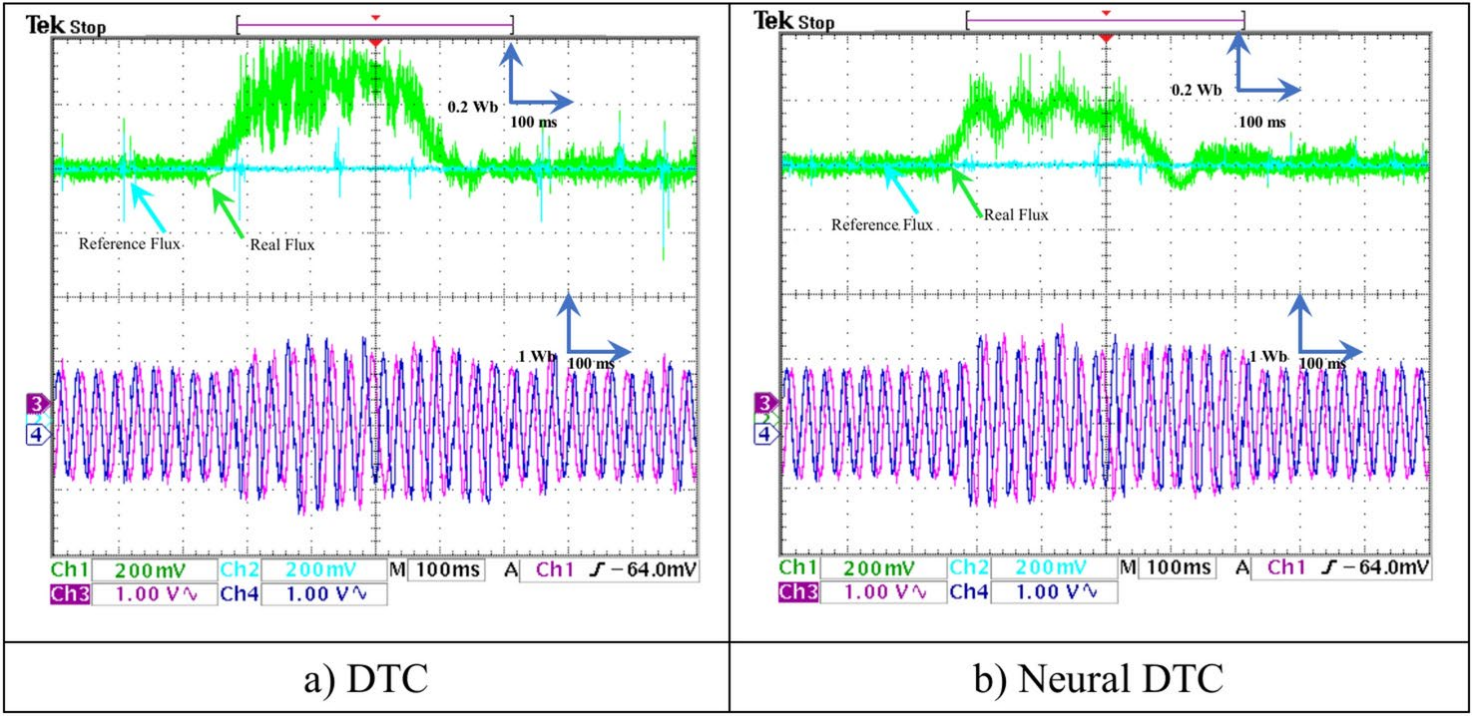
Stator flux and its components for 1480 rpm (no-load and with speed variation).

**Fig. 25:**
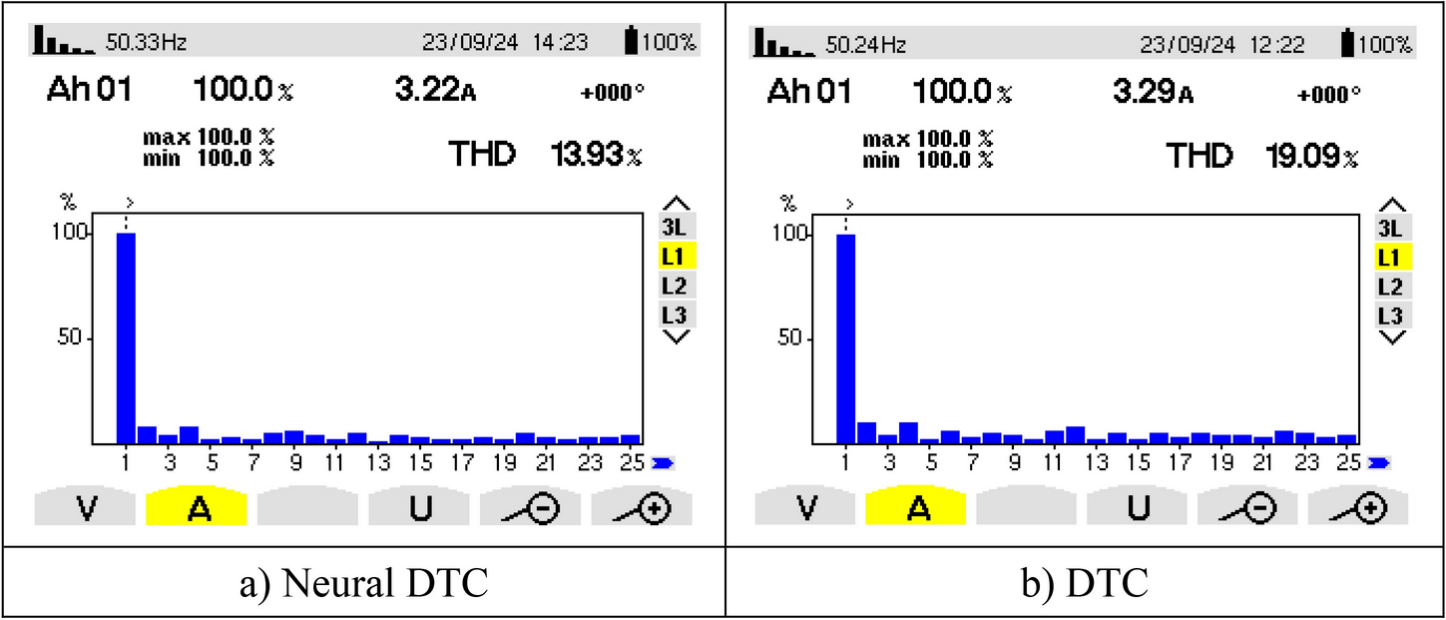
THD of current (The second experimental test).

In this test, the efficacy of the two approaches is analyzed and verified in the case of a SCIM speed change from 1000 to 1480 rpm at no-load conditions. The results of this test are presented in Figs. 22,23,24,25 for this test, a 500 mV scale with a gain of 0.01 was used for torque measurements, a 500 mV scale with a gain of 0.0001 for speed measurements, and a 200 mV scale with a gain of 0.1 for flux measurements. The scales and gains were adjusted in this test to prevent saturation of the dSPACE system.

Figure 22 shows both the torque and the motor speed of both approaches. Regarding the torque, it follows the reference value in this test with the effect of a speed change from 1000 to 1480 rpm. It is observed that the torque value rises to the maximum value and then gradually decreases, with the presence of fluctuations in the case of using the three approaches. However, there are fewer ripples for the 6 sectors neural DTC technique. In the case of the speed, it is observed that all approaches follow the reference with varying overshoots and response times. The 6-sector neural DTC technique demonstrates a fast response time and lower overshoot compared to the 6-sector DTC technique.

Figure 23 shows both the stator currents of the SCIM in test 2, where it is noted that the change in SCIM speed is accompanied by a change in current. From this figure, it is observed that the current is affected by the speed change, and its value increases with the rise in rotational speed during a transient period. The current waveform at 1480 rpm indicates the variation in rotational frequency.

Figure 24 represents the experimental change in both flux and current for the two commands. In Fig. 24, the stator flux (1 div = 0.2 Wb) and its components (1 div = 1 Wb) are depicted for a speed variation from 1000 to 1480 rpm. From this figure, it is observed that the flux exceeds the limit value within a limited time period, and this overshoot and the ripples are relatively smaller in the case of the 6 sectors neural DTC technique compared to the DTC technique. The same applies to the stator flux components, which show an increase in value within the same period while maintaining a sinusoidal waveform.

Figure 25 represents the THD value obtained experimentally using the two controls. Through this figure, the THD value was estimated at 19.09% and 13.93% for both the traditional DTC technique and the proposed approach, respectively. So, the proposed approach reduced the THD value significantly experimentally compared to the conventional DTC strategy, as this reduction was estimated at a rate of 27.03% compared to the traditional approach. It is noted from Fig. 25 that the amplitude value of the fundamental signal (50 Hz) was 3.29 A and 3.22 A for both the conventional DTC technique and the proposed approach, respectively. Therefore, the amplitude value is larger if the conventional DTC approach is used compared to the proposed approach. So the amplitude value is negative in the proposed approach in this test. This negativity can be overcome in the future by using the neural strategy with other strategies such as genetic algorithms.

Table 5 represents a study of the effect of changing the amplitude value of the fundamental signal (50 Hz) and current THD for the two controls during the first and second experimental tests. From this table, it is noted that the amplitude value did not change in the traditional DTC approach, as its value remained constant in the second test. However, in the case of the proposed approach, it is noted that the amplitude value increased slightly in the second test compared to the first test. The difference in the amplitude value in the case of using the proposed approach between the first and second tests was estimated at 0.01 A. Table 5 shows that the THD value increased in the second experimental test compared to the first experimental test for two controls. This increase was estimated at 32.79% and 26.56% for both the traditional DTC technique and the proposed approach, respectively. Therefore, the traditional approach had the greatest effect on THD compared to the proposed approach. These results highlight the effectiveness of the proposed approach in improving the THD value and raising the quality of the current, making it a promising and reliable solution in the field of control in the future.

**Table 5:** Study of the effect of changing the amplitude of the fundamental signal (50 Hz) and the THD current between the second and first experiments.

		DTC approach	DTC-NN approach
Amplitude of fundamental signal (50 Hz)	**Test 1**	3.29	3.21
	**Test 2**	3.29	3.22
	**Test 2 – Test 1**	00	+ 0.01
	**Ratios (%)**	00	0.31
THD (%)	**Test 1**	12.83	10.23
	**Test 2**	19.09	13.93
	**Test 2 – Test 1**	+ 6.26	+ 3.7
	**Ratios (%)**	32.79	26.56

### Load test

**Fig. 26:**
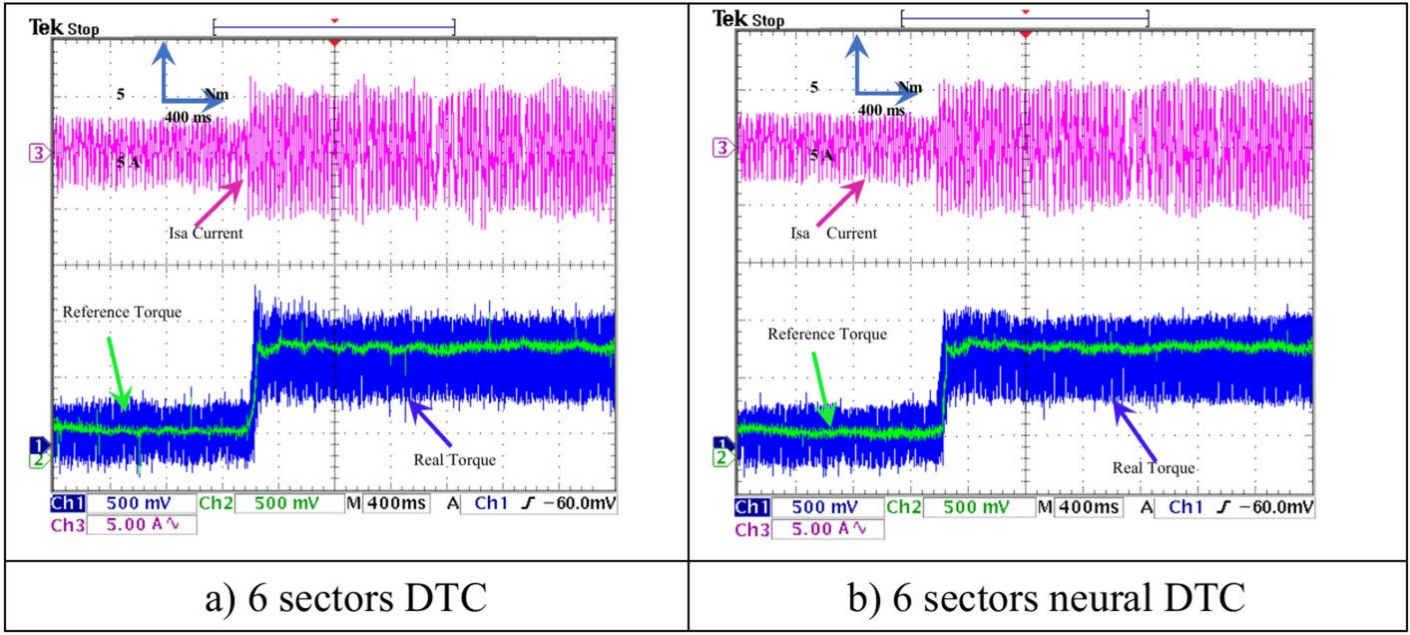
Torque and stator current *I*_*sa*_ (under load).

**Fig. 27:**
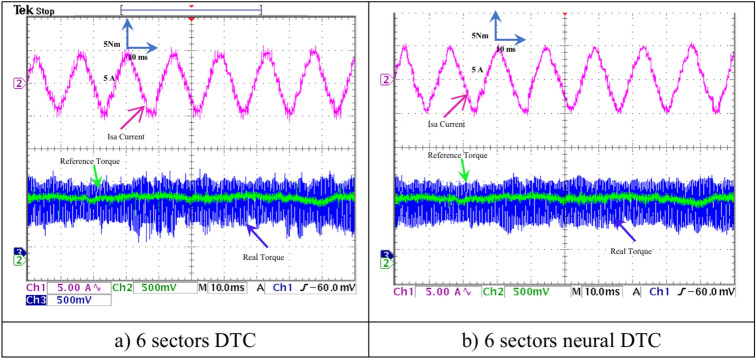
Zoom: Torque and stator current *I*_*sa*_ (under load).

**Fig. 28:**
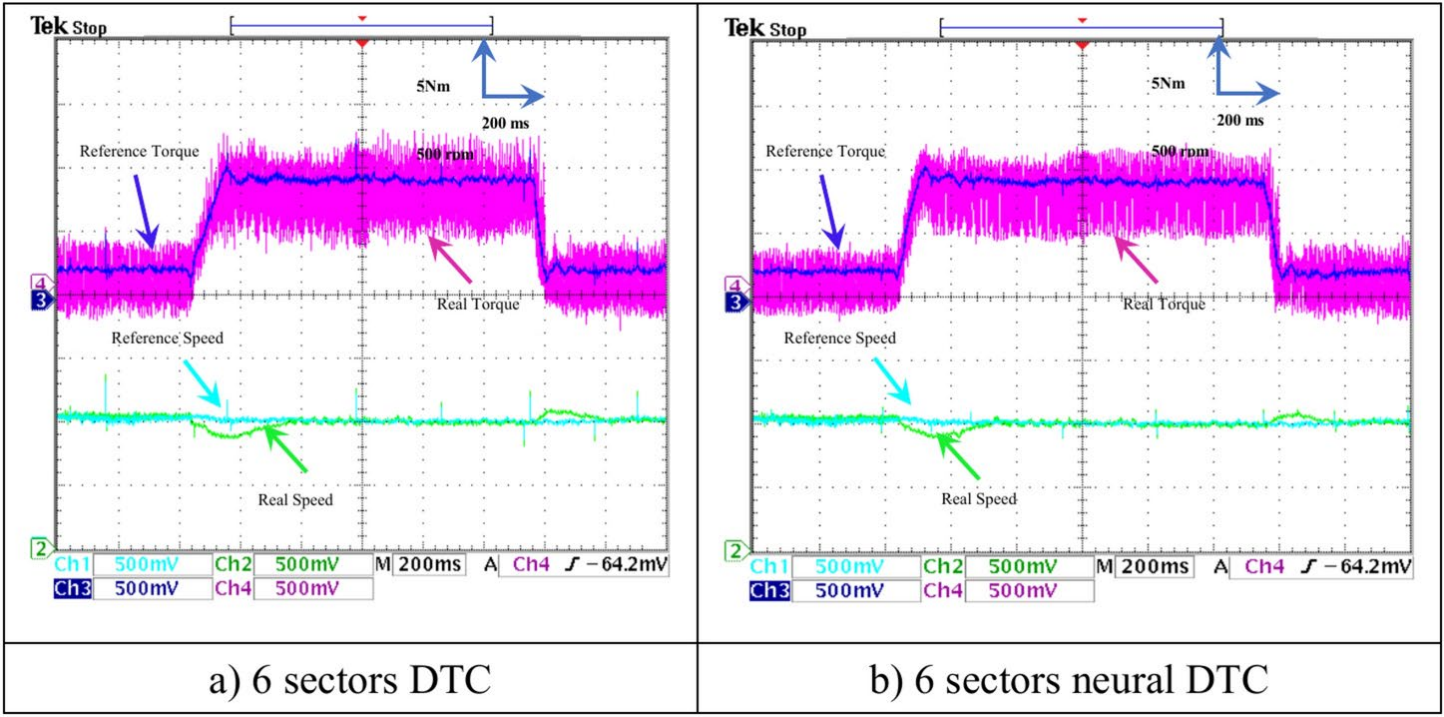
Rotor speed and torque (under load).

**Fig. 29:**
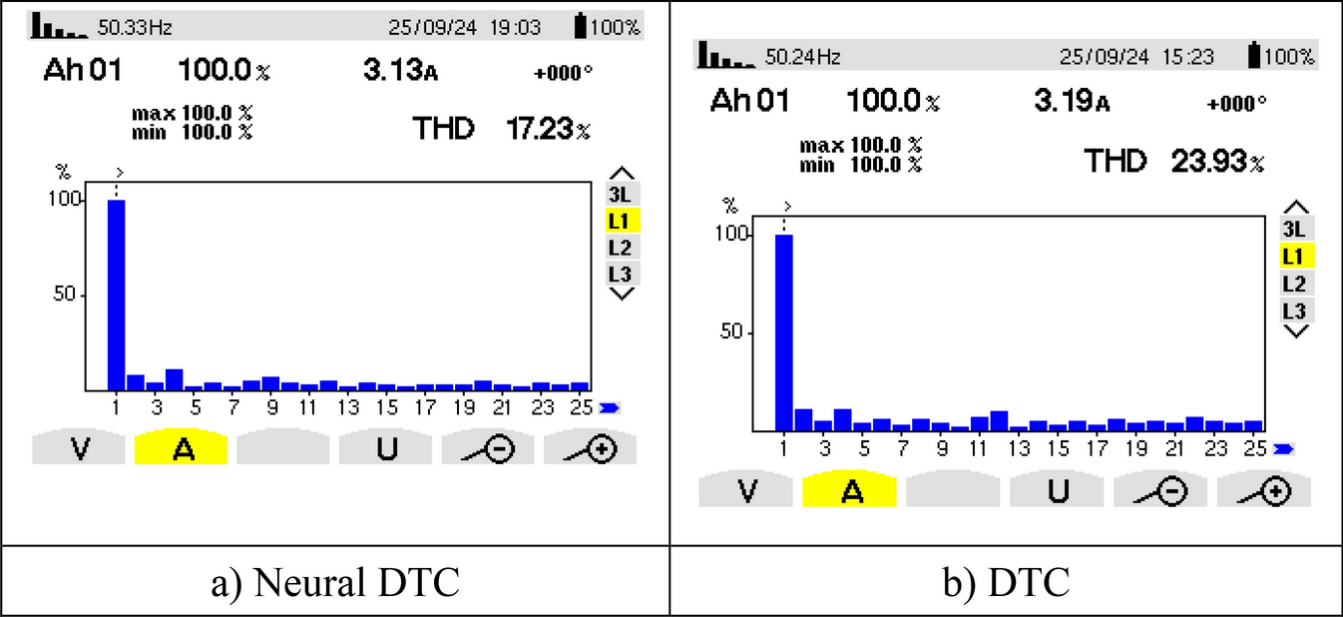
THD of current (Test 3).

In this section, the results of the load test performed on the experimental platform are shown for the two control methods. Figures 26,27,28,29 show the experimental results obtained from the DTC technique and the neural DTC technique of the SCIM fed by a two-level inverter. The SCIM is driven at a speed of 1000 rpm. The sampling time is set to *Te* = 10^–4^ s using the Euler method. The reference flux is 0.8 Wb.

In this test, a 500 mV scale with a gain of 0.01 was used for torque measurements, and a 500 mV scale with a gain of 0.0001 was used for speed measurements. The scales and gains were adjusted in this test to prevent the saturation of the dSPACE system and to highlight the changes, particularly in terms of speed.

Figure 26 shows the torque (1 div = 5 N.m) and the current *I*_*sa*_ (1 div = 5 A) of the SCIM at the moment when the load is applied. It can be observed from this figure that the SCIM torque closely follows the reference torque for the three 6-sector DTC technique and 6-sector neural DTC technique. Additionally, the electrical current increases at the moment the load is applied, with this augment corresponding to the applied load (the value of the applied resistant torque). Furthermore, there are torque and current ripples, as shown in Fig. 27, which are more pronounced in the case of the 6-sector DTC technique compared to the 6-sector neural DTC technique. These applied results are consistent with those presented in the simulation section, indicating the validity of the previously presented results and the superiority of the neural DTC technique.

The SCIM speed and torque are illustrated in Fig. 28. From this figure, it is observed that the speed (1 div = 500 rpm) closely follows the reference for 6 sectors neural DTC technique. In the case of using DTC technique, the value of response time and overshoot are greater compared to the proposed neural DTC approach when applying load. These results convincingly confirm the simulation results and demonstrate that the neural DTC technique is superior to the DTC technique, particularly in terms of reducing torque fluctuations.

Figure 29 represents the amplitude value and THD of current for two controls. From this figure, the proposed approach gave an amplitude of 3.13 A and a THD value of 17.23%. The amplitude value and THD value for the conventional approach were estimated at 3.19 A and 23.93%, respectively. Through these values, the proposed approach significantly reduces the THD value compared to the traditional DTC approach. This reduction in THD value was estimated at 28% compared to the conventional approach. However, the proposed approach offered less scope than the traditional DTC technique, which is negative. This negativity can be attributed to the characteristics of the neurological approach used, as this negativity can be overcome in the future.

Table 6 shows a study of the effect of the value of the current THD and the amplitude of the fundamental signal (50 Hz) for two controls in the case of the first and third experimental tests. This table shows that the THD value for two controls increased in the third experimental test compared to the first experimental test. This increase in the THD value was estimated at 46.39% and 40.63% for both the traditional DTC approach and the proposed approach, respectively. Therefore, the proposed approach provided a lower impact rate than the traditional DTC technique, which highlights its high performance and strength, which makes it a promising solution in the future. The amplitude value in the two control conditions decreased in the third experimental test compared to the first experimental test. This decrease in the amplitude value was estimated at 3.04% and 2.49% for both the DTC approach and the proposed approach, respectively. Through these percentages, the proposed approach provided a lower impact rate than the DTC technique. Therefore, it can be said that the value of both amplitude and THD were slightly affected if the proposed approach was used compared to the 6-sector DTC technique. These results highlight the effectiveness and efficiency of the proposed approach, making it a promising solution for the future.

**Table 6:** Study of the effect of both the fundamental signal amplitude (50 Hz) and the THD current between the third and first experimental tests.

		DTC approach	DTC-NN approach
Amplitude of fundamental signal (50 Hz)	**Test 1**	3.29	3.21
	**Test 3**	3.19	3.13
	**Test 3 – Test 1**	-0.10	-0.08
	**Ratios (%)**	-3.04	-2.49
THD (%)	**Test 1**	12.83	10.23
	**Test 3**	23.93	17.23
	**Test 3 – Test 1**	+ 11.10	+ 7
	**Ratios (%)**	46.39	40.63

## Conclusions

This experimental work deals with the proposed 6-sector neural DTC approach to control SCIM using real tools, where the performance and efficiency were compared with the DTC technique. The neural DTC technique relies on the use of NNs, which makes it highly performant and highly robust, as simplicity, DR, and ease of realization are among the most prominent features of the 6-sector neural DTC technique. These features make the neural DTC technique an effective solution in future propulsion and traction systems. The proposed approach provides fewer torques and current ripples compared to the DTC technique in the simulation and experimental. Also, the six-sector neural DTC demonstrated better experimental response time than the 6-sector DTC approach, as the response time was equal to 380 ms and 400 ms for both the neural DTC and DTC algorithms, respectively. Therefore, the use of NNs is considered effective in addressing the defects of the DTC technique, and this is highlighted by the experimental results. The main contributions of this work lie in improving the DTC technique properties and increasing the mechanical performance of SCIM, making it a promising solution for the future.

Future work will focus on continuing the experimental work on the DTC technique of SCIM using other strategies, such as synergetic control and fractional calculus techniques while comparing the experimental results with other related works.

## Data Availability

Data available on request from the authors. The datasets used and/or analysed during the current study available from the corresponding author on reasonable request. In the event of communication, the fist author (Habib Benbouhenni, E-mail: ha-bib.benbouhenni@enp-oran.dz) will respond to any inquiry or request.

## Abbreviations

NN: Neural Network
THD: Total Harmonic Distortion
DTC: Direct Torque Control
FL: Fuzzy Logic
SFV: Stator Flux Vector
FOC: Filed-Oriented Control
PI: Proportional-Integral Controller
PWM: Pulse Width Modulation
SMC: Sliding Mode Control
HC: Hysteresis Comparator
BC: Backstepping Control
SCIM: Squirrel Cage Induction Machine
IM: Induction Machine
SV: Switching Vector
ST: Switching Table
SVV: Stator Voltage Vector
FLC: Fuzzy Logic Controller
MM: Mathematical Model
DR: Dynamic Response
MPC: Model Predictive Control
GWO: Grey Wolf Optimization
SSE: Steady-State Error
